# A Systematic Review of Artificial Intelligence Models for Time-to-Event Outcome Applied in Cardiovascular Disease Risk Prediction

**DOI:** 10.1007/s10916-024-02087-7

**Published:** 2024-07-19

**Authors:** Achamyeleh Birhanu Teshale, Htet Lin Htun, Mor Vered, Alice J. Owen, Rosanne Freak-Poli

**Affiliations:** 1 School of Public Health and Preventive Medicine, Monash University, Melbourne, VIC Australia; 2 Department of Data Science and AI, Faculty of Information Technology, Monash University, Clayton, VIC Australia; 3 Stroke and Ageing Research, Department of Medicine, School of Clinical Sciences at Monash Health, Monash University, Melbourne, VIC Australia; 4 Department of Epidemiology and Biostatistics, Institute of Public Health, College of Medicine and Health Sciences, University of Gondar, Gondar, Ethiopia

**Keywords:** Cardiovascular Disease, Prediction, Machine Learning, Deep Learning, Artificial Intelligence

## Abstract

Artificial intelligence (AI) based predictive models for early detection of cardiovascular disease (CVD) risk are increasingly being utilised. However, AI based risk prediction models that account for right-censored data have been overlooked. This systematic review (PROSPERO protocol CRD42023492655) includes 33 studies that utilised machine learning (ML) and deep learning (DL) models for survival outcome in CVD prediction. We provided details on the employed ML and DL models, eXplainable AI (XAI) techniques, and type of included variables, with a focus on social determinants of health (SDoH) and gender-stratification. Approximately half of the studies were published in 2023 with the majority from the United States. Random Survival Forest (RSF), Survival Gradient Boosting models, and Penalised Cox models were the most frequently employed ML models. DeepSurv was the most frequently employed DL model. DL models were better at predicting CVD outcomes than ML models. Permutation-based feature importance and Shapley values were the most utilised XAI methods for explaining AI models. Moreover, only one in five studies performed gender-stratification analysis and very few incorporate the wide range of SDoH factors in their prediction model. In conclusion, the evidence indicates that RSF and DeepSurv models are currently the optimal models for predicting CVD outcomes. This study also highlights the better predictive ability of DL survival models, compared to ML models. Future research should ensure the appropriate interpretation of AI models, accounting for SDoH, and gender stratification, as gender plays a significant role in CVD occurrence.

**Supplementary Information:**

The online version contains supplementary material available at 10.1007/s10916-024-02087-7.

## Introduction

Cardiovascular diseases (CVD) cause 32% of all global deaths [1]. A confluence of environmental, genetic, social, and physiological factors leads to the development of CVD incidence, hospitalisation, and mortality. Early detection of these factors and appropriate interventions are recommended approaches for decreasing CVD burden and impact. Various multivariable prediction models exist for early detection of CVD risk [2], including the latest risk estimation tools like pooled cohort equations (PCE) [3], Systematic COronary Risk Evaluations (SCORE, SCORE2, and SCORE2 for older people) [4, 5], and Predicting Risk of cardiovascular disease EVENTs (PREVENT) [6].

However, due to the availability of big data and advancing technology, machine learning (ML) and deep learning (DL) prediction models, the two subfields of artificial intelligence (AI), have increasingly being utilised. Several studies have indicated that ML and DL models surpass traditional multivariable models in predicting CVD risk and specific events such as stroke [7]. These AI models exhibit enhanced discrimination and risk stratification abilities.

**Fig. 1:**
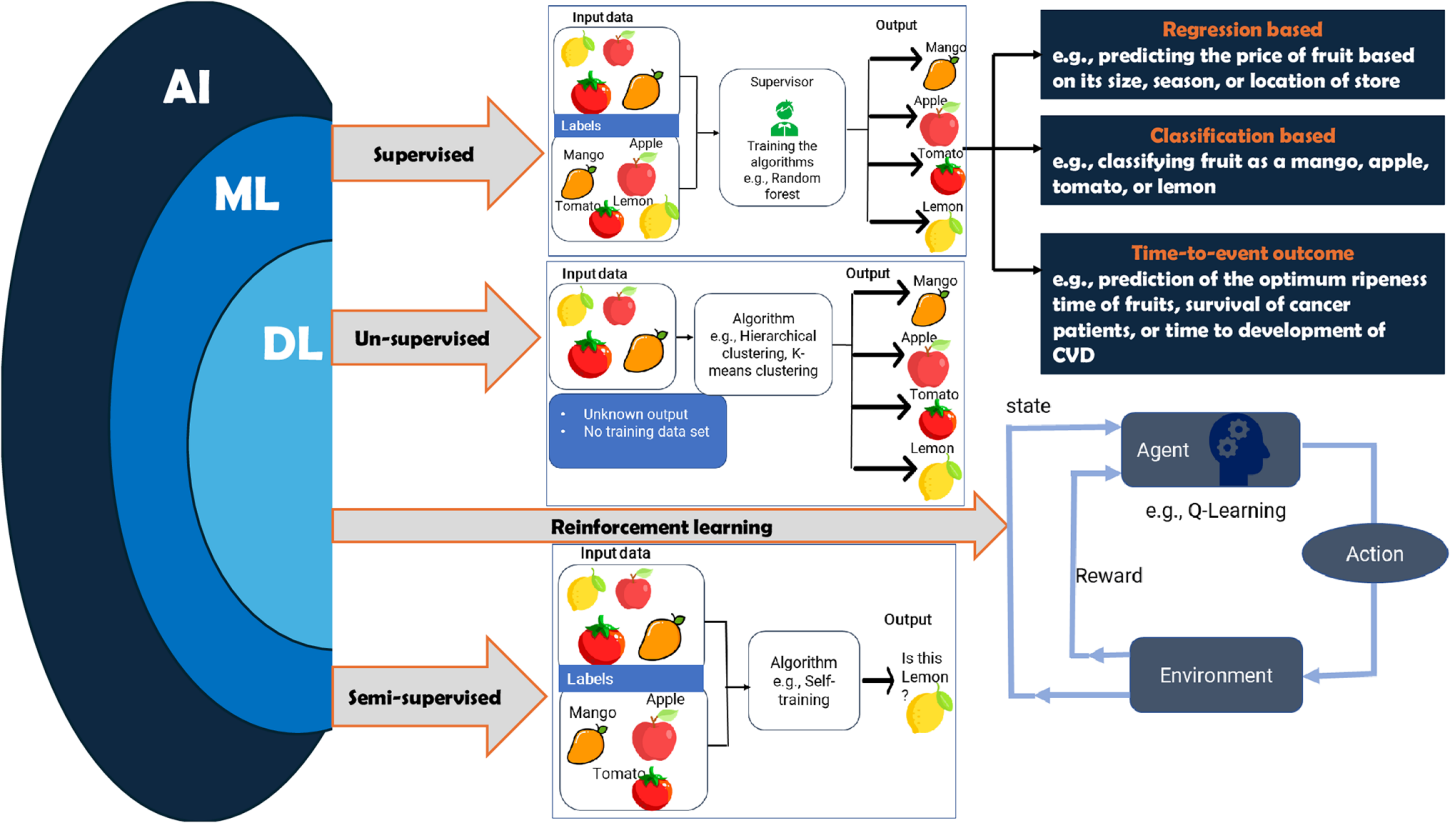
Overview of machine learning and deep learning models

ML uses algorithms and statistical models to analyse and draw inferences from patterns in the data. DL is a subset of ML that uses artificial neural networks. As illustrated in Fig. 1, there are primarily four types of ML and DL [8, 9]; supervised, unsupervised, semi-supervised, and reinforcement learning (RL). Supervised ML algorithms have been utilised for future risk prediction. They require training using labelled data, that is data that contains inputs and correct corresponding outputs. Depending on the type of the outcome variable that is available for the learning phase, regression and classification algorithms (including binary, multi-class, multi-level, and imbalanced classification) are commonly employed. Regression algorithms predict continuous variables while classification algorithms determine the likelihood that a certain event will occur. Unsupervised ML algorithms (such as anomaly detection, clustering) use unlabelled data and are intended to find groups/clusters of similar characteristics without human supervision. Semi-supervised ML combines the features of supervised and unsupervised ML approaches, i.e., utilises both labelled and unlabelled data. RL algorithms interact with an environment to learn the optimal behaviour to maximise the overall reward.

ML models have been in existence since 1957. The perceptron, which laid the foundation for supervised ML models and artificial neural networks, was one of the earliest neural network models. Since then, ML has passed several important milestones: the development of decision trees in the 1960s, support vector machines (SVM) in the 1990s, random forest in 2001, DL models in the 2010s, large language models such as ChatGPT in 2022, and many others recently [10]. These models are supervised ML algorithms for classification and regression, and are applied for predicting or forecasting chronic diseases, including CVD risk [11]. However, ML and DL algorithms for survival prediction were not widely used until Random Survival Forest (RSF) was developed by Ishwaran et al. in 2008 [12]. In particular, survival AI prediction algorithms, which estimate the time until a health outcome occurs, have not received as much attention as classification and regression ML algorithms [13]. Currently, various AI models for right-censored data are gaining popularity, even though they are predominantly used for predicting cancer patient survival outcomes [14–17]. Survival forest models [16, 17], NonLinear Cox proportional hazard (Cox PH) model (also known as DeepSurv model), and Neural Multi-Task Logistic Regression (NMTLR) [14–16] are among the commonly utilised models. Some studies have also employed algorithms such as CoxTime and Cox-CC [14].

Numerous systematic reviews on AI-based CVD prediction have been conducted [18–21]; yet they primarily focus on classification-based models. For instance, Baashar et al.‘s research assessed the effectiveness of ML and DL in CVD prediction through network meta-analysis [20], covering 17 studies from 2016 to 2021 and suggesting that DL might yield better results than ML. Nonetheless, a systematic review that succinctly summarise ML and DL models for right-censored data is still lacking. The justification for exploring AI models for right-censored data stems from the unique nature of survival outcomes. Unlike regression and classification problems, survival outcome must account for two components during model training: the follow-up time, which is continuous, and the event status, indicating whether a specific event has occurred, such as CVD, represented as a binary outcome.

Additionally, previous risk prediction models, including the latest multivariable prediction models and AI-based models mentioned above, primarily focus on standard modifiable risk factors of CVD, demographics (age and sex/gender), and lifestyle factors (particularly smoking). This means that social determinants of health (SDoH), defined as the social and environmental circumstances in which people grow, live, work, worship, and age, have been overlooked in disease prediction models [22], including CVD [18, 23]. For example, only race in the PCE [3] and social deprivation in the PREVENT tool [6] are incorporated when predicting CVD. Similarly, AI-based risk prediction models consider only a limited number of SDoH variables, like race, income, and occupation [18]. SDoH are detailed in the Healthy People 2030 framework using five domains, namely, economic stability, education quality and access, social and community context, neighborhood and built environment, and healthcare access and quality [24]. Using the Healthy People framework as a foundation, our umbrella review [25] demonstrated that SDoH have a major role in development of CVD. In general, disparities in SDoH give rise to health inequalities, which are systematic discrepancies in the opportunities people need to attain optimal health.

**Fig. 2:**
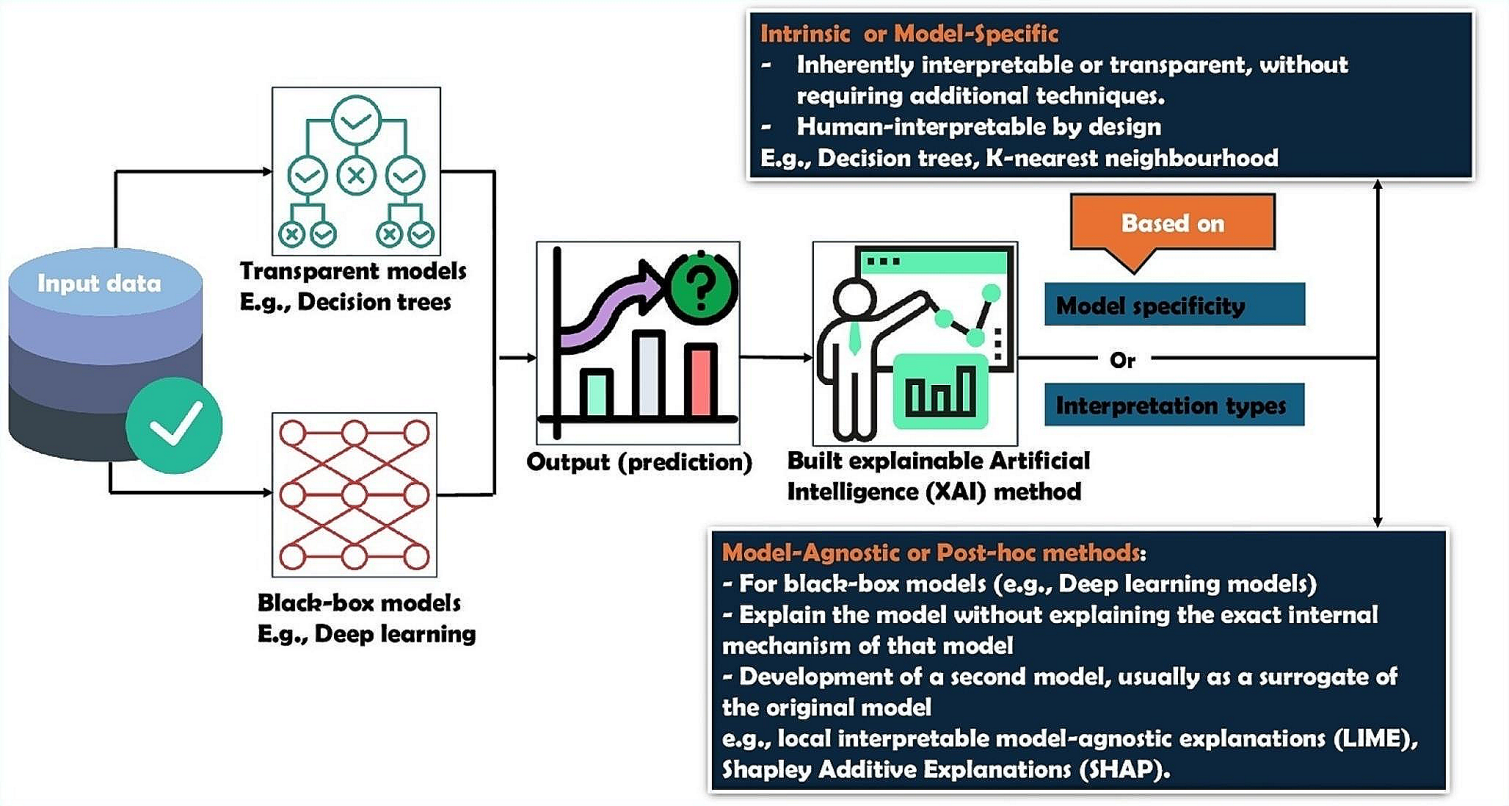
Overview of eXplainable AI approaches

It is also important to focus on the explainability of the AI models to improve confidence in their application. These are called eXplainable AI (XAI) techniques and, as shown in Fig. 2, these can be model-specific (use the structure of the model itself, e.g., built in feature importance measures in ensemble models) or model-agnostic (provide post-hoc explanations e.g., Local Interpretable Model-agnostic Explanations (LIME), Shapley Additive exPlanations (SHAP)) [26]. However, these techniques have limited application for survival ML and DL methods. New XAI techniques for survival models such as Survival SHAP (SurvSHAP), survival neural additive model (SurvNAM), and survival LIME (SurvLIME) are currently gaining attention but are only used for explaining some algorithms [27, 28]. XAI in general, aims to increase user trust in a model to different stakeholders: (1) those with model expertise (e.g., ML experts, researchers); and (2) those without (clinicians, patients). However, the explainability of time to event AI models has been less explored.

Moreover, various systematic reviews have been conducted on AI-based CVD prediction [18–21]; however, they have all focused-on classification-based models (models designed for classification problems). For example, research conducted by Baashar et al. evaluated the efficacy of ML and DL in the prediction of CVD through network meta-analysis [20]. The study encompassed 17 studies spanning from 2016 to 2021, concluding that DL may offer more favorable outcomes than ML in predicting CVD.

Therefore, this systematic review aims to (1) investigate AI models for survival prediction employed in predicting CVD; (2) indicate whether XAI is applied for interpreting the models; and (3) examine whether the identified AI models account for SDoH as well as gender stratification.

## Methods

### Registration and Reporting

The study protocol was registered in the International Prospective Register of Systematic Reviews (PROSPERO CRD42023492655). The Preferred Reporting Items for a Systematic Review and Meta-analysis (PRISMA) statement is used for reporting [29] (Table S1).

### Eligibility Criteria

Studies were deemed eligible if they intended to predict CVD outcomes using AI methods for survival prediction. There were no restrictions based on country, study design, language, and study period (Table 1). Grey literature (including conference abstracts), case reports, letters, editorials, and reviews were not eligible. AI models based on simulation and imaging/text data are ineligible as they do not use structured population-level data.

**Table 1:** Key items for framing the aim, search strategy, and study inclusion and exclusion criteria

Domain	Description
Population	Adult population (age ≥ 18 years)
Intervention (Models)	Machine learning and deep learning predictive models for time to event outcome
Comparator	Not applicable
Outcomes	One or more of the following cardiovascular disease outcomes; (1) cardiovascular disease or major adverse cardiovascular event (MACE); (2) cardiovascular disease subtypes such as coronary heart disease, angina, myocardial infarction, heart failure, cerebrovascular disease (hemorrhagic and ischemic stroke), heart disease; or (3) hospitalisation or mortality due to cardiovascular disease or subtypes of cardiovascular diseases.
Time	No restriction
Setting	Both community and institution (e.g., hospital).

### Information Sources and Search Strategy

We carried out a comprehensive search using five electronic databases, from their inception to December 21, 2023: Embase via Ovid, Scopus, Web of Science, IEEE Xplore, and Ovid Medline. Further studies were identified by a manual search using Google Scholar, and through backward and forward reference searching using Web of Science. Various terms related to CVD, AI methods, and risk prediction were utilised (Table 2), linked through Boolean and adjacency (or proximity) operators. The comprehensive search terms used in Ovid Medline are available in the supplementary file (Table S2).

**Table 2:** Summary of keywords/search terms per each concept

No.	Categories	Keyword
1.	Cardiovascular disease (including mortality or hospitalisation)	Cardiovascular disease, coronary heart disease, ischemic heart disease, angina, atrial fibrillation, major cardiovascular event, myocardial infarction, heart failure, congestive heart failure, heart disease, cerebrovascular disease/event, stroke, ischemic stroke, hemorrhagic stroke, and peripheral arterial disease
2.	Artificial intelligence	Artificial intelligence, machine learning, deep learning, random survival forest, Extra Survival Trees, survival ensembles, survival support vector machine, Multi-Task Logistic Regression, DeepSurv, Non-Linear Cox proportional hazard model, CoxTime, CoxCC, probability mass function, Nnet-survival, DeepHit, DeepHitSingle, Piecewise Constant Hazard model, Discrete-Time Models, Continuous-Time Models, Neural network, survival neural network, deep neural survival networks
3.	Risk prediction	Prediction, risk assessment, prognosis, predict, predictive modeling, detect, identify, identification, detection, risk stratification
4.	Right censored time to event out come	Time to event, right censored, survival analysis, survival data, censoring

### Study Selection and Data Extraction

Identified records from databases were exported to Endnote Version 20 and then to ASReview [30] and Covidence [31]. Following deduplication, eligible articles at the title and abstract stage were selected using ASReview. Full text screening was done using Covidence. Using the data extraction sheet prepared based on the 11 CHecklist for critical Appraisal and data extraction for systematic Reviews of prediction Modelling Studies (CHARMS) domains [32], data were extracted. Two reviewers (ABT and HLH) selected eligible studies and undertook data extraction, resolving conflicts through discussion (full-text review: proportionate agreement = 96%, Cohen’s κ = 0.92).

### Data Synthesis

Characteristics of studies were summarised based on items from the CHARMS statement and our specific aims. If a study used more than one ML or DL algorithm, we reported the prediction performance measure for the best performing algorithm. ML and DL models were compared with each other and with the standard Cox PH model. Utilised SDoH variables, based on the Healthy People 2030 framework, were reported. XAI methods employed were described. However, due to variations in the study population, the endpoint description, the different ML and DL algorithms utilised, and the variety in the types and numbers of variables, the prediction performance of the models was not pooled (i.e., meta-analysis was not conducted).

### Assessment of Risk of Bias

To evaluate the risk of bias (RoB), we used the Prediction Model Risk of Bias Assessment Tool (PROBAST) [32] with four domains: participant selection; predictors; outcome; and analysis, and different signaling questions per each domain. Using the PROBAST, we also assessed applicability using three domains: participant, predictors, and outcome. Two authors (ABT and HLH) assessed RoB independently and any disagreements were resolved by discussion.

## Results

### Screening Result

**Fig. 3:**
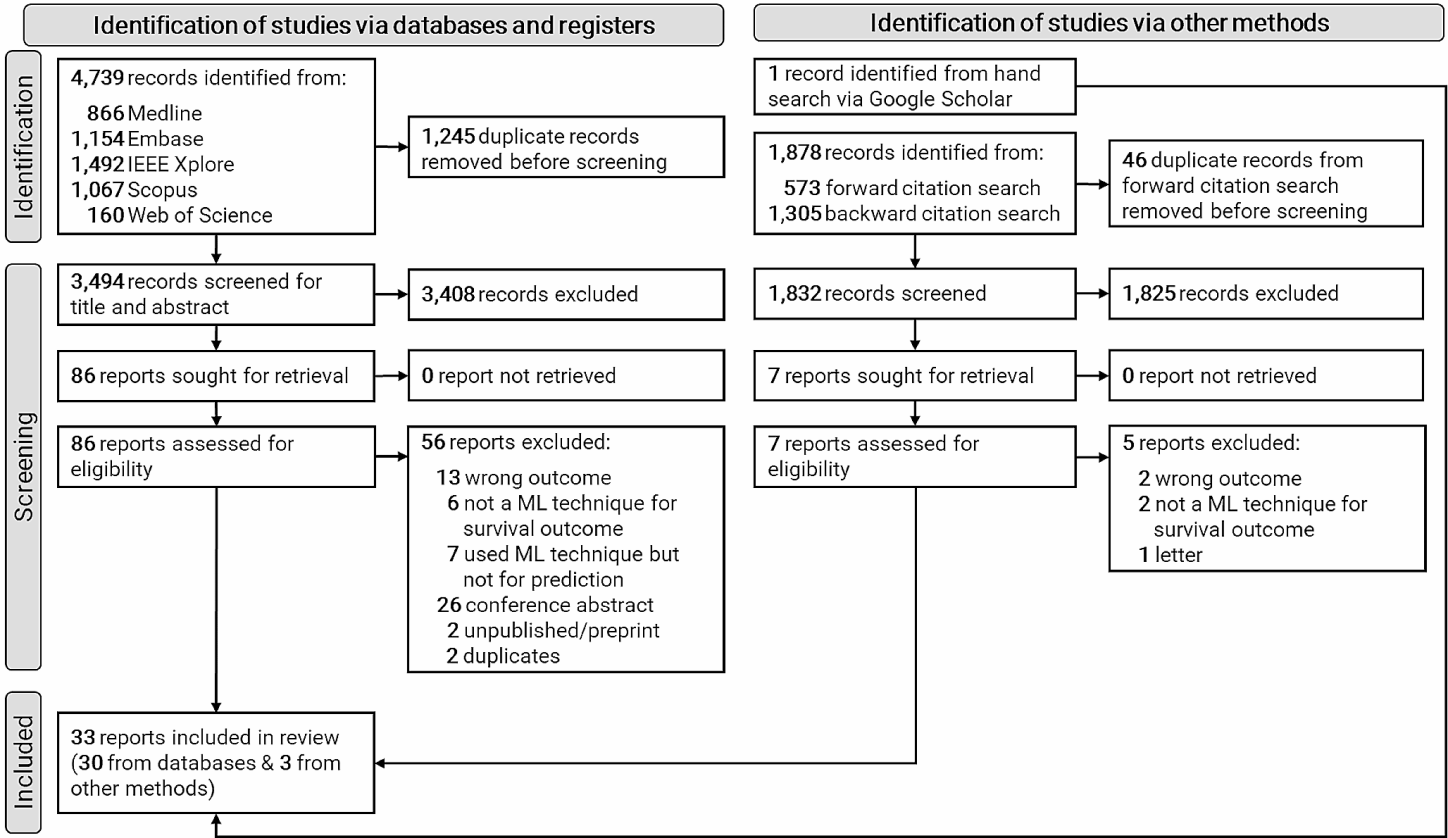
PRISMA flow diagram showing the study selection process

Out of a total of 4,739 studies retrieved through database searching, 86 were eligible for a full-text review. Thirty-three studies in total, 30 studies [33–62] from database searching and three studies [63–65] from other sources, qualified for inclusion in this study (Fig. 3). The studies that were excluded during the full-text review are provided in the supplementary file (Table S3).

### Characteristics of the Included Studies

The majority of studies were published in 2023 (*n* = 16/33; 48.5%) and originated from the United States (*n* = 13/32; 40.6%; one study did not report the country). Approximately, 39.4% (*n* = 13/33) of the studies used a sample size of 10,000 or more. Two studies focused exclusively on one gender (one on men and the other on women), whilst the majority of studies analysing both genders had a higher percentage of women (50% and above) (Table S4).

### Follow-up Time and Incidence of Cardiovascular Diseases

**Fig. 4:**
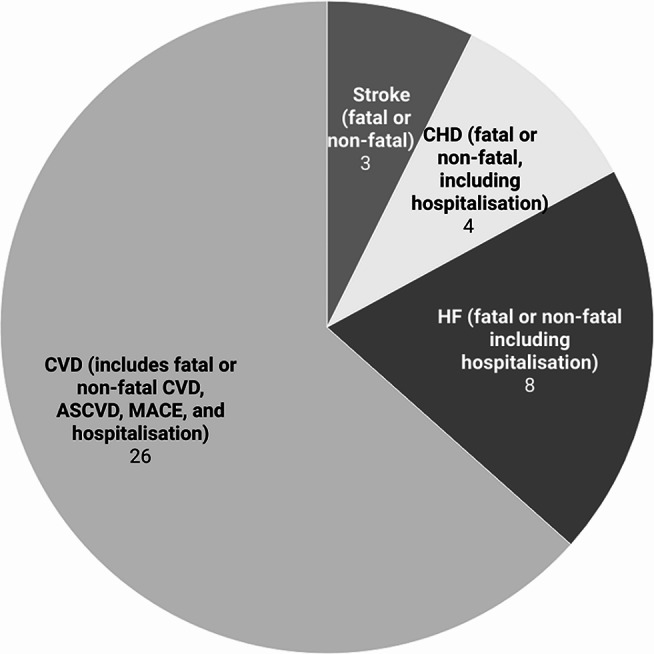
Number of studies based on predicted cardiovascular diseases outcomes. ASCVD: Atherosclerotic cardiovascular disease; CHD: Coronary heart disease; CVD: Cardiovascular disease; HF: Heart failure; and MACE; Major adverse cardiovascular events. Note: Since one study can incorporate more than one outcome, the sum total reported here exceeds the total number of included studies

The mean or median follow-up time ranges from 4.4 years to 25.03 years in studies of community-dwelling people, and 4.3 months to 8.05 years in studies of institutionalised-people (Table S4). Various CVD outcomes, with their definitions and corresponding ICD codes detailed in Table S4, were identified (Fig. 4). The incidence of CVD outcomes ranged from 2.1% (CVD-related mortality) to 43.7% (MACE) (Table S4).

### Model Related Characteristics

#### Employed ML and DL Models

**Fig. 5:**
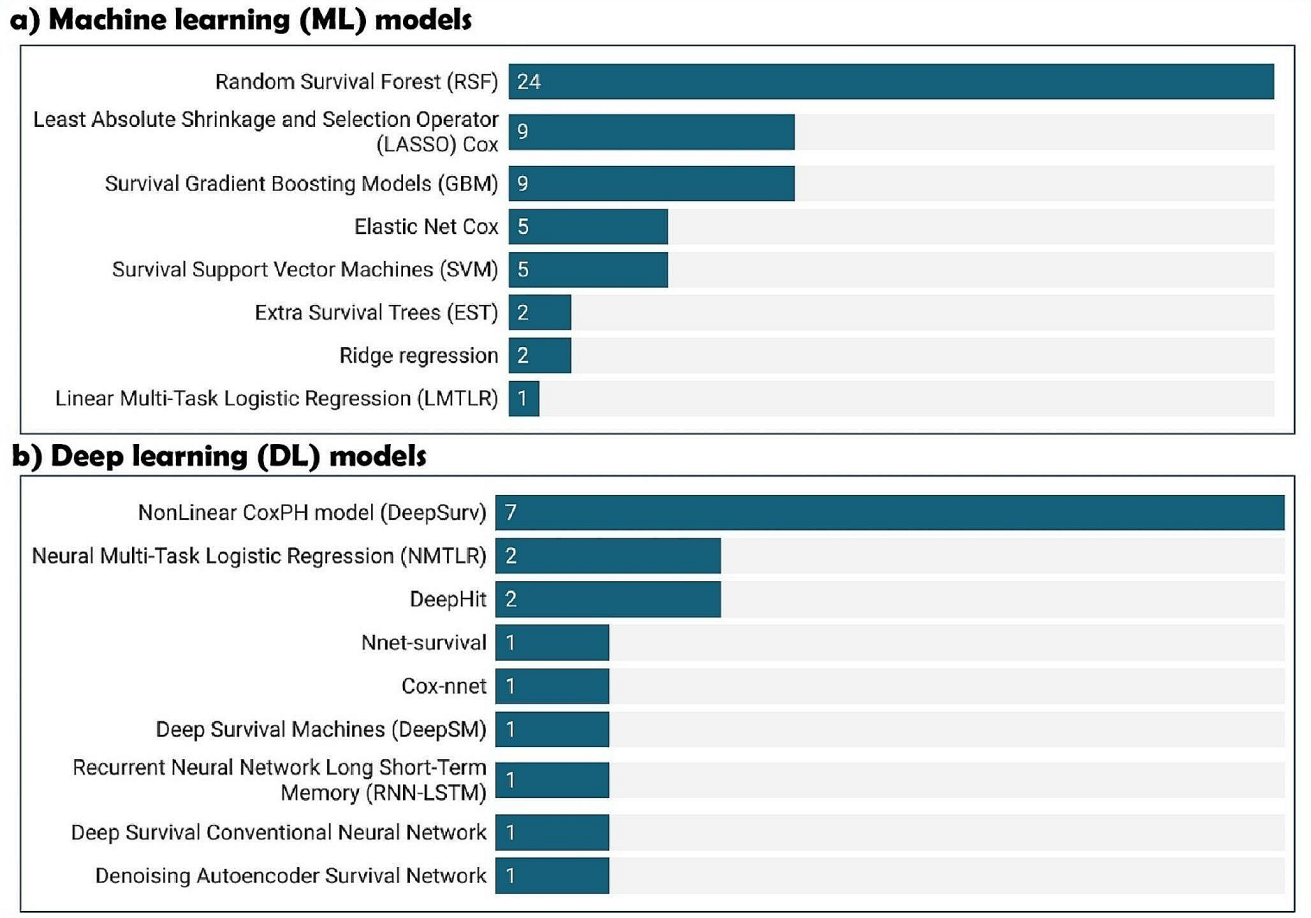
Identified ML and DL models. Since a single study could utilise multiple ML and/or DL models, the total number of studies presented here exceeds 33 (the total number of studies included)

Eight ML and nine DL models were utilised, with Fig. 5 presenting the names of each model and the number of studies that employed them, and Table S5 providing further details.

### Best Performing ML and DL Models

To evaluate the predictive performance, studies utilised C-index, area under the curve (AUC), and Brier score or calibration plot. In addition, some studies also explored other measures such as decision curve analysis. These performance evaluation metrics are presented in Table S5. The mean C-index (standard deviation) was 0.79 (0.069) for ML models, 0.82 (0.061) for DL models, 0.81 (0.144) for Penalised Cox, 0.80 (0.058) for RSF, 0.79 (0.032) for DeepSurv, and 0.77 (0.055) for survival Gradient Boosting Models (GBM) (Table 3).

**Table 3:** Descriptive statistics of predictive performance (C-index/area under the curve) by ML and DL algorithms

Machine learning and deep learning models (number of studies^f^)	Mean C-index (Standard deviation)^g^	Median C-index^g^	Min	Max	Inter Quartile Range
Random survival forest (*n* = 23)	0.80 (0.058)	0.80	0.65	0.92	0.77-0.0.83
DeepSurv (*n* = 10)	0.79 (0.032)	0.79	0.74	0.85	0.77–0.81
Survival Gradient Boosting Models (*n* = 7)	0.77 (0.055)	0.75	0.72	0.87	0.72–0.81
Other deep learning models^a^ (*n* = 6)	0.85 (0.081)	0.83	0.76	0.96	0.79–0.94
Penalised Cox^b^ (*n* = 4)	0.81 (0.144)	0.81	0.66	0.93	0.69–0.93
Machine learning models^c^ (*n* = 34)	0.79 (0.069)	0.79	0.65	0.96	0.75–0.83
Deep learning models^d^ (*n* = 16)	0.82 (0.061)	0.80	0.74	0.96	0.78–0.84

^a^Includes DeepHit, Neural Multi-Task Logistic Regression, Recurrent Neural Network Long Short-Term Memory, and deep survival conventional neural network

^b^Includes LASSO and Elastic Net Cox models

^c^Includes Random Survival Forest, survival Gradient Boosting Models, and Penalised Cox models

^d^Includes Deepsurv, Neural Multi-Task Logistic Regression, Recurrent Neural Network Long Short-Term Memory, and Deep Survival Conventional Neural Network

^f^Total number of models may differ from total number of included studies, because some studies reported for men and women separately or fitted a model based on race and studies may not report the C-index or area under the curve quantitatively

^g^Area under the curve if the study did not report C-index

**Fig. 6:**
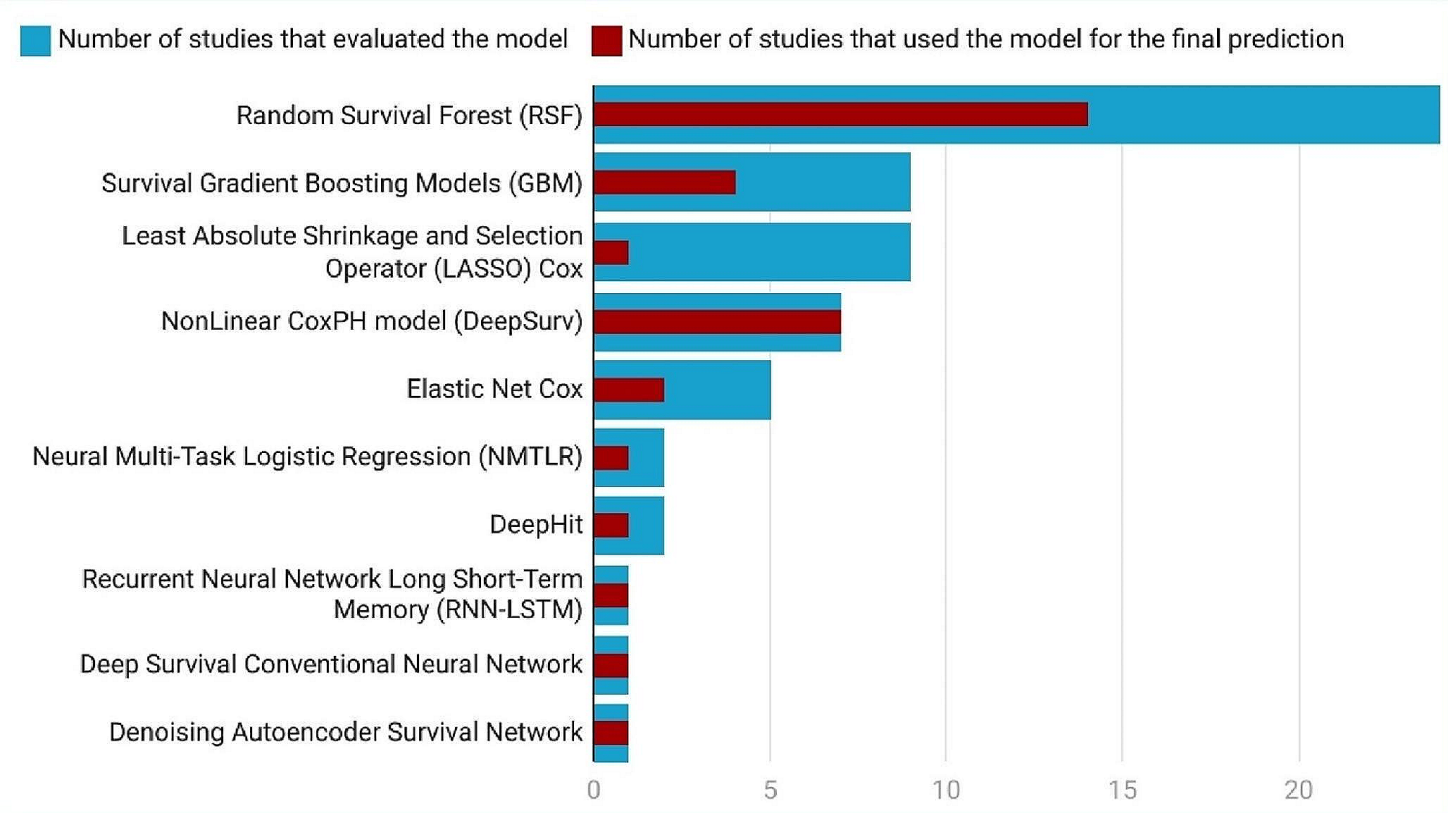
Number of studies that evaluated the prediction model and used it for their final prediction

Eight studies only used one ML [35, 37, 39, 41, 47, 50, 57, 58] and four studies only used one DL [34, 36, 43, 59] algorithm to predict CVD. The 26 studies [33–37, 39–46, 48, 49, 52–57, 59, 62–65] that compared ML and DL models with the Cox PH model revealed that ML and DL models were better in predicting CVD. Nine studies [33, 38, 51–53, 55, 60, 62, 65] among 18 studies [33, 38, 42, 44, 46, 48, 49, 51–55, 60–65] that compared the RSF with other models, selected RSF as the best performing model. Six studies [35, 37, 39, 41, 50, 57] used RSF without comparison with other ML or DL models. Among the seven studies [38, 44, 55, 56, 63–65] that compared the survival GBM with other models, the boosting models were better in two studies [56, 64]. In another two studies [47, 58], the boosting method was used to predict CVD without comparing with other models. All of the five studies [38, 44, 45, 56, 63] that evaluated Elastic Net Cox compared the model with other models, and in the two studies [45, 63] Elastic Net Cox was the best performing model for CVD prediction. In the nine studies [33, 45, 49, 52, 53, 60–62, 65] that evaluated LASSO-Cox, all compared the model with other models, it was in only one study [61] that LASSO-Cox was selected as the best performing model. As for the DL models, the DeepSurv model was the best performing model in all the five studies [34, 40, 43, 46, 48, 49, 54] that compared the model with other models. In the other two studies [34, 40], DeepSurv was not compared with other ML or DL models. Some studies also selected NMTLR [42, 46], DeepHit [51, 52], and denoising autoencoder survival network [44] as best performing models in predicting CVD. Others [36, 59] examined Recurrent Neural Network Long Short-Term Memory and Deep Survival Conventional Neural Network, however, without comparing with any other ML or DL models (Fig. 6 and Table S5).

### Comparison of ML and DL Models

Among the eight studies [42, 44, 46, 48, 49, 51, 52, 54] that compared ML and DL models together, DL models were better in predicting CVD risk in seven studies [42, 44, 46, 48, 49, 51, 54]. Four studies [46, 48, 49, 54] compared the DeepSurv model with other ML and DL models and all four found this model to be the best for predicting CVD outcomes (Table 4 and Table S5).

**Table 4:** Selected models, based on their performance, among studies that compared machine learning and deep learning models together

Author and year	Selected best model	Deep Learning models	Machine learning models
Feng 2022 [42]	Neural Multi-Task Logistic Regression	Neural Multi-Task Logistic Regression	Random survival forest and Linear Multi-Task Logistic Regression
Gao 2023 [44]	Denoising autoencoder Survival network	Denoising autoencoder Survival network	Elastic Net Cox, Gradient Boosted Survival, Support Vector Machine, and Random survival forest
Hathaway 2021 [46]	DeepSurv/NonLinear CoxPH model	Deepsurv/NonLinear CoxPH model and Neural Multi-Task Logistic Regression	Random survival forest and Support Vector Machine
Kim 2023 [48]	DeepSurv/NonLinear CoxPH model	Deepsurv/NonLinear CoxPH model and Deep Survival Machines (DeepSM)	Random survival forest
Lin 2023 [49]	DeepSurv/NonLinear CoxPH model	Deepsurv/NonLinear CoxPH model	LASSO-Cox and Random survival forest
Ren 2022 [54]	DeepSurv/NonLinear CoxPH model	Deepsurv/NonLinear CoxPH model	Random survival forest
Morris 2023 [51]	DeepHit	DeepHit	Random survival forest and Penalised cox proportional hazards
Nguyen 2023 [52]	Random Survival Forest	DeepHit	Random survival forest and LASSO-Cox

### Utilised XAI Techniques

The 25 studies interpreted their model using different approaches (Table 5 and Table S5). Out of those studies, four [49, 52, 63, 64] used more than one method. Eight studies [33, 34, 37, 40, 42, 44, 58, 60] provided no model interpretation.

**Table 5:** Model interpretation methods

Model interpretation technique utilised	Number of studies
Feature importance (e.g., permutation (majority), Mean Decrease Gini, mean of the minimal depth of the maximal subtree)	14 [35, 39, 41, 46, 48–50, 52–54, 56, 62, 65]
Shapley Additive exPlanations (SHAP)	6 [47, 51, 52, 61, 63, 64]
Partial dependence plots (PDPs)	4 [43, 55, 63, 64]
Machine learning derived/simplified risk score (including nomogram)	3 [45, 49, 57]
Temporal Importance Model Explanation (TIME)	1 [52]
Layer-wise Relevance Propagation (LRP)	1 [59]
Component wise gradient boosting coefficients	1 [38]
Contribution of features using weighted ratio (WR)	1 [36]

### Model Validation

All studies internally validated their prediction model using either train-test splitting or using resampling methods such as k-fold cross-validation and Bootstrapping. However, only six studies [40, 46, 56, 57, 59, 65] externally validated their prediction model. The commonly employed models were RSF and DeepSurv (Table S5).

### Gender Stratification

Of 31 studies that used gender in their prediction models, only six studies [34, 36, 39, 40, 53, 59] performed gender-stratified prediction. RSF and DL models such as DeepSurv were most popular (Table S5).

### Number of Predictors and Feature Selection Methods

The number of candidate predictors ranged from 7 to 950. Mostly the number of candidate predictors was greater than 50 (*n* = 13/33; 39.4%). The number of final predictors used ranged from 3 to 613, with the majority (*n* = 13/33; 39.4%) incorporating 21 to 50 variables (Table S5). In 22 studies, variable selection was not performed. Of those studies that performed variable selection prior to training, two used RSF, two used LASSO-Cox, one used stepwise forward selection, and one used Elastic Net Cox. Additionally, five studies used more than one variable selection method (Table S5).

### Types of Predictors

**Fig. 7:**
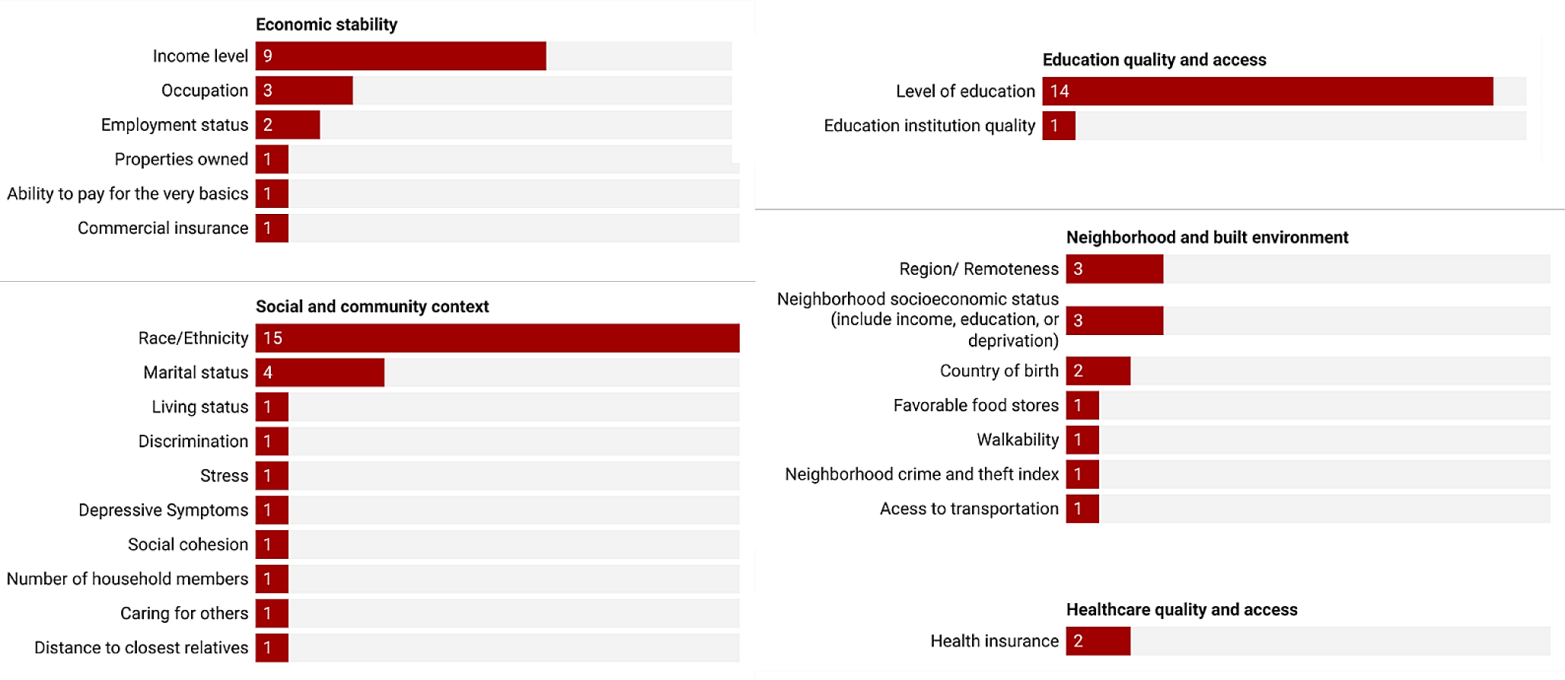
Number of studies that incorporated social determinants of health variables for predicting cardiovascular disease

Included studies used a wide range of risk factors such as standard modifiable cardiovascular risk factors, demographic factors, imaging features, biomarkers, variables related to sleep and diet, environmental chemicals, and SDoH variables (Table S5). Regarding our study’s interest in SDoH, at least one SDoH was included by 20 studies [33, 34, 38–40, 42, 46, 47, 50–53, 55, 57, 58, 60–63, 65] as a candidate variable. Of these 20 studies, all except for two [53, 57] incorporated at least one SDoH as a final predictor to train the model. However, only two studies [51, 58] employed a wide range of SDoH variables from the Healthy People 2030 framework. The most frequently considered SDoH variables were race/ethnicity, level of education, and income (Fig. 7 and Table S5).

### Models Based on Population

Eighteen studies [33, 34, 36, 37, 39, 40, 46, 50–53, 55, 56, 59, 61–63, 65] were conducted among the general population (relatively healthy adults) (Table S4). Half of the studies [33, 37, 39, 50, 52, 53, 55, 62, 65] employed RSF, after comparison [33, 52, 53, 55, 62, 65] or used RSF as the only model [37, 39, 50]. Six studies [34, 36, 40, 46, 51, 59] used DL models in which three used DeepSurv [34, 40, 46], three did comparison with other models [40, 46, 51], and three did not do comparison [34, 36, 59]. In three studies, Elastic Net Cox [63], LASSO-Cox [61], and Survival GBM [56] were the best performing models (Table S5).

The remaining 15 studies [35, 38, 41–45, 47–49, 54, 57, 58, 60, 64] were conducted among institutionalised populations or individuals with specific medical conditions (Table S4). Nine studies [35, 38, 41, 45, 47, 57, 58, 60, 64] used ML models, five studies [35, 38, 41, 57, 60] utilised RSF (after comparison [38, 60] or as the only model [35, 41, 57]), three studies [47, 58, 64] survival GBM (after comparison [64] or as the only model [47, 58]), and one study [45] utilised Elastic Net Cox (after comparison with other models) for their final prediction. Six studies [42–44, 48, 49, 54], all after comparison with other models, used DL models. Four of the studies [43, 48, 49, 54] used the DeepSurv model and the other two used NMTLR [42] and denoising autoencoder survival network [44] (Table S5).

#### Models Based on Types of Variables

##### Models Incorporated Imaging Features

11 studies [33, 35, 38, 44, 46, 48, 50, 56, 57, 62, 65] included image features, with standard modifiable risk factors, SDoH factors, or other factors, in their prediction models (Table S5). Seven of them [33, 38, 50, 57, 62, 65] utilised RSF, after comparing with other models [33, 38, 62, 65] or as the only model [35, 50, 65]. No studies compared RSF with DL models; instead, they compared RSF with other ML models. One study [56] selected survival GBM, after comparing with other ML models. Three studies utilised DL models, two DeepSurv [46, 48] and one denoising autoencoder survival network [44]. These three models were compared with ML learning models (such as RSF) and DL models (such as NMTLR).

##### Models Accounted for SDoH

Among the 18 studies that included at least one SDoH (detailed above) as their final predictor (Table S5), nine studies utilised RSF, because RSF was the best performing model in seven studies [33, 38, 52, 55, 60, 62, 65] or used as the only model [39, 50]. Two studies employed Elastic Net Cox [63] and LASSO-Cox [61] since they were the best performing models as compared to other models. Two studies [47, 58] utilised survival GBM without comparing the model with other ML or DL models. Five models utilised DL models; three studies used DeepSurv [34, 40, 46], two studies NMTLR [42], and one study DeepHit [51]. All these studies compared their DL model with other ML models (mostly with RSF) and/or other DL models.

### Software and Related Information

Twelve studies [33, 41, 44, 45, 50, 53, 55, 57, 58, 60, 62, 65] used R software (packages: *glmnet*, *ranger*, *randomForestSRC*, *mlr3*, *mlr3proba*). Meanwhile, nine studies [34, 36, 38, 47, 48, 51, 56, 61, 64] utilised Python software (packages: *scikit-survival*, *PySurvival*, *pycox*). Studies also used more than one software, for instance, 11 studies [35, 37, 39, 40, 42, 43, 46, 49, 52, 54, 63] used R and Python. The specifics of the missing data management and hyperparameter tuning methods, along with the libraries and packages (including source codes) utilised in the studies are presented in Table S6.

#### Risk of Bias

Among all 33 studies, 15 [33, 38, 45, 49–51, 53, 54, 57–59, 61, 62, 64, 65] had low (RoB); 14 [34–37, 39–42, 44, 46, 52, 55, 56, 63] had high RoB; and four [43, 47, 48, 60] had uncertain RoB. As for applicability, 19 studies [34, 38–40, 42, 43, 45, 47, 49, 53, 55–58, 60–62, 64, 65] had low applicability concerns and 14 [33, 35–37, 41, 44, 46, 48, 50–52, 54, 59, 63] had high applicability concerns (Table 6).

**Table 6:** Risk of bias and applicability assessment

Author, Year	Risk of Bias				Applicability			Overall	
	1. Participants	2. Predictors	3. Outcome	4. Analysis	1. Participants	2. Predictors	3. Outcome	Risk of Bias	Applicability
Ambale-Venkatesh, 2017	**+**	**+**	**+**	**+**	**+**	**-**	**+**	**+**	**-**
Barbieri, 2022	**+**	**+**	**+**	**-**	**+**	**+**	**+**	**-**	**+**
Bauer, 2023	**+**	**+**	**+**	**-**	**+**	**-**	**+**	**-**	**-**
Blanchard, 2022	**+**	**+**	**+**	**-**	**+**	**-**	**+**	**-**	**-**
Brester, 2023	**+**	**+**	**+**	**-**	**+**	**-**	**+**	**-**	**-**
Chhoa, 2023	**+**	**+**	**+**	**+**	**+**	**+**	**+**	**+**	**+**
Chun, 2021	**+**	**+**	**+**	**-**	**+**	**+**	**+**	**-**	**+**
Deng, 2023	**+**	**+**	**+**	**-**	**+**	**+**	**+**	**-**	**+**
Duan, 2024*	**-**	**+**	**+**	**+**	**-**	**+**	**+**	**-**	**-**
Farhadian, 2021	**?**	**+**	**+**	**-**	**+**	**+**	**-**	**-**	**-**
Feng, 2022	**+**	**+**	**+**	**-**	**+**	**+**	**+**	**-**	**+**
Gandin, 2023	**?**	**+**	**+**	**?**	**+**	**+**	**+**	**?**	**+**
Gao, 2023	**+**	**+**	**+**	**-**	**+**	**+**	**-**	**-**	**-**
Garcia-Carretero, 2019	**+**	**+**	**+**	**+**	**+**	**+**	**+**	**+**	**+**
Hathaway, 2021	**+**	**+**	**+**	**+**	**+**	**-**	**+**	**-**	**-**
Jain, 2021	**+**	**+**	**+**	**?**	**+**	**+**	**+**	**?**	**+**
Kim, 2023	**+**	**+**	**+**	**?**	**+**	**+**	**-**	**?**	**-**
Lin, 2023	**+**	**+**	**+**	**+**	**+**	**+**	**+**	**+**	**+**
Mauger, 2023	**+**	**+**	**+**	**+**	**+**	**+**	**-**	**+**	**-**
Moreno-Sánchez, 2023	**+**	**+**	**+**	**+**	**+**	**+**	**+**	**+**	**+**
Morris, 2023	**+**	**+**	**+**	**+**	**+**	**+**	**-**	**+**	**-**
Nguyen, 2023	**+**	**+**	**+**	**-**	**+**	**+**	**-**	**-**	**-**
Qian, 2023	**+**	**+**	**+**	**+**	**+**	**+**	**+**	**+**	**+**
Ren, 2022	**+**	**+**	**+**	**+**	**+**	**+**	**-**	**+**	**-**
Rigdon, 2019	**-**	**+**	**+**	**-**	**+**	**+**	**+**	**-**	**+**
Sabovcik, 2022	**+**	**+**	**+**	**-**	**+**	**+**	**+**	**-**	**+**
Segar, 2019	**+**	**+**	**+**	**+**	**+**	**+**	**+**	**+**	**+**
Segar, 2021	**+**	**+**	**+**	**+**	**+**	**+**	**+**	**+**	**+**
Stabellini, 2023	**+**	**+**	**+**	**+**	**+**	**+**	**+**	**+**	**+**
Sung, 2019	**+**	**+**	**+**	**+**	**+**	**+**	**-**	**+**	**-**
Turchin, 2023	**+**	**+**	**?**	**+**	**+**	**+**	**+**	**?**	**+**
Wang, 2023	**+**	**+**	**+**	**+**	**+**	**+**	**+**	**+**	**+**
Zhuang, 2022	**+**	**+**	**+**	**+**	**+**	**+**	**+**	**+**	**+**

* The accepted manuscript (pre-proof) was found during the search and was published in January 2024

+ indicates low risk of bias/low concern regarding applicability

- indicates high risk of bias/high concern regarding applicability

? indicates unclear risk of bias/unclear concern regarding applicability

## Discussion

### Main Findings

**Fig. 8:**
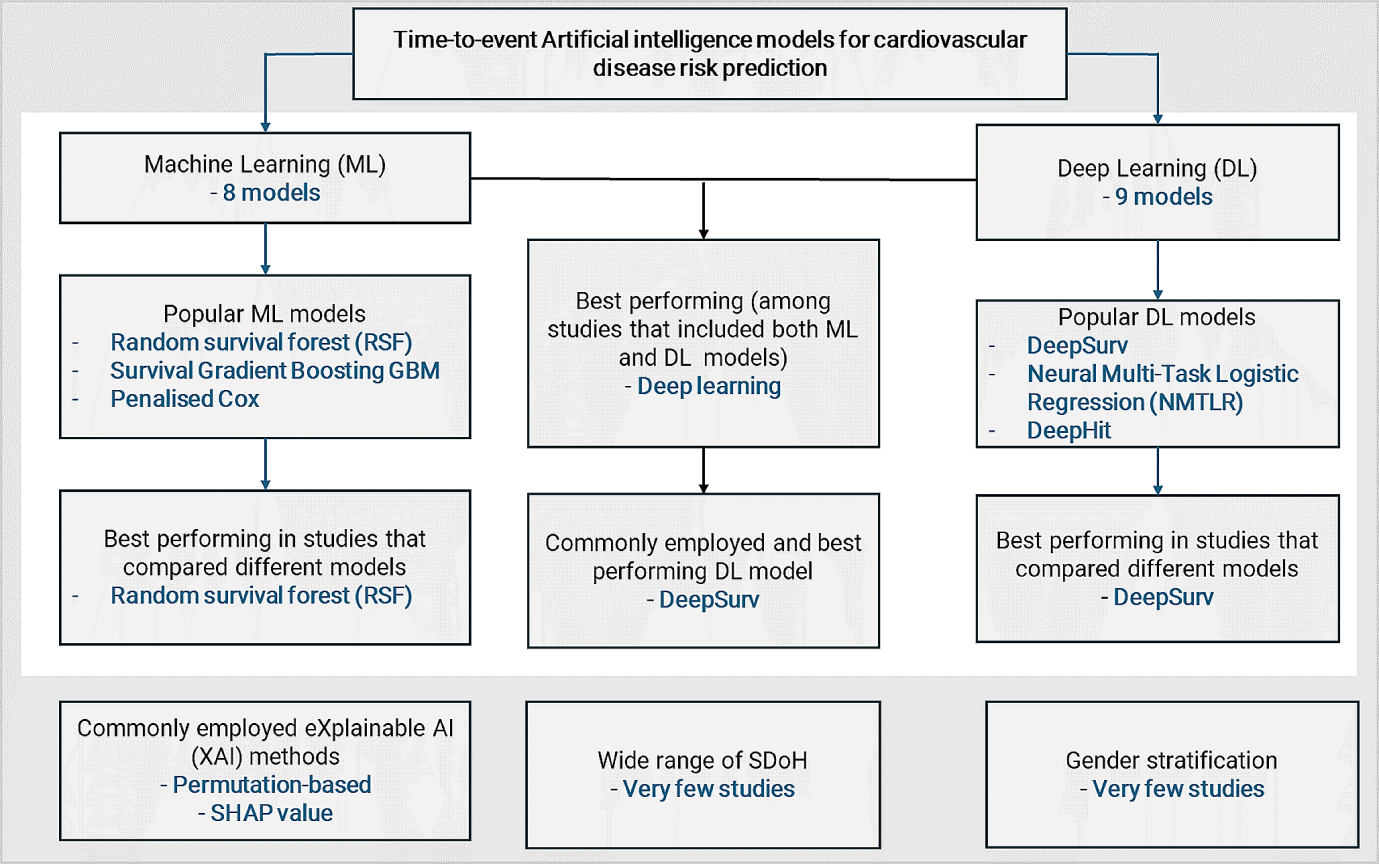
Summary of the principal findings

To enhance clarity, our principal findings are depicted in Fig. 8. A variety of ML and DL models for survival prediction in CVD were identified. The popular ML methods were RSF, survival GBM, and Penalised Cox models. These three ML models also performed best at predicting time to CVD occurrence, when compared to numerous other ML models considered in the included studies. Regarding DL models, the models that were utilised most frequently were DeepSurv, NMTLR, and DeepHit. These three DL models had better performance, compared with other DL or ML models. Permutation based feature importance and SHAP values were the predominant XAI methods utilised for explaining the models. While a variety of variables were incorporated to predict CVD, there was a noticeable lack of consideration for a wide range of SDoH variables. Additionally, prediction modeling with gender stratification was rarely explored.

### AI Models for Survival Prediction and Year of Publication

Our systematic review revealed ML and DL models for survival prediction are increasingly gaining attention, while nearly all studies were published in 2019 or afterwards, half were published in 2023. This is not surprising since the packages in R and Python (e.g., *mlr3proba* package in R, and *pycox*, *scikit-survival*, *PySurvival* Python packages) for survival prediction using AI models became available in 2019 or later [66–69].

### ML Models for Survival Outcome in CVD Prediction

The most utilised ML model was RSF. Our finding corroborate a scoping review on applications of ML in predicting survival outcomes, which identified RSF as the most frequently utilised model [13]. RSF has become a well-developed and user-friendly model, since its introduction by Ishwaran et al. in 2008 [12]. RSF is effective at handling complex interactions, has built-in variable importance measures, and is robust to overfitting [70]. The other plausible explanation is due to the availability of numerous open-source packages in standard software such as R and Python for appropriate training of RSF [66–69].

The next commonly applied ML models were survival GBM and Penalised Cox models. This may be at least partly explained by survival GBM considers non-linear interactions, has a high reported prediction accuracy, has greater ease of interpretability, and has automatic variable selection [71]. The availability of packages in R and Python (effectively open-source) makes the model easily trainable and accessible. For instance, survival GBM can be efficiently trained using the newly developed Python package, scikit-survival [69]. Both the RSF model and survival GBM are ensemble models that combine the decisions from several baseline models to improve the overall performance and robustness [72]. The Penalised Cox models are commonly used because they are important for penalising and provide a parsimonious model [73]. They are easier to apply (having a few, maximum two, parameters to tune). Finally, a few studies also utilised survival SVM, Linear Multi-Task Logistic Regression (LMTLR), and Extra survival trees in predicting CVD.

### DL Models for Survival Outcome in CVD Prediction

Different DL models were also utilised to predict CVD. The DeepSurv model was mostly utilised. The other models were the NMTLR and DeepHit. These models are also commonly applied in oncologic studies [15, 74–76]. Most of these models can be well-trained using the two important Python packages, *PySurvival* and *Pycox*, which have been popular since 2019 [66–68]. Denoising autoencoder survival network, Recurrent Neural Network Long Short-Term Memory, and Deep Survival Conventional Neural Network [66, 68] were also utilised by some of the studies.

### Best Performing ML and DL Models for Survival Outcome in CVD Prediction

Compared to the standard Cox proportional hazards model, both ML and DL models have demonstrated superior performance, in terms of discriminative ability and calibration. Another review has also shown that ML models outperform conventional methods in predicting health outcomes [13]. This may be due to the limited capability of the standard Cox model to handle high-dimensional datasets and its reliance on a linear relationship assumption, which are often not met.

The most frequently selected ML models (based on their prediction performance) were ensemble methods (RSF and survival GBM). RSF and survival GBM are ensemble models that are known to have superior prediction performance because they are drawn from several baseline learners [72]. However, this finding might also be a result of RSF and survival GBM models being considered in many of the included studies. In three studies, Penalised Cox-models were also selected as the best preforming. Penalised Cox models reduce overfitting, handle multicollinearity (particularly the Elastic Net Cox), enhance interpretability, and automate variable selection by shrinking less important predictors’ coefficients to zero [77].

As for DL models, in almost all studies, DeepSurv was selected as the best performing model. Our finding corroborates multiple individual studies on the survival prediction of cancer patients, demonstrating that DeepSurv surpasses alternative methods in predictive accuracy [14, 15, 76]. DeepSurv computes complex and non-linear features without a priori selection or domain expertise and is helpful for personalised risk prediction, even better than other linear and non-linear survival methods [78]. Notably, DeepSurv was also popular and, therefore, available for comparison.

### Comparison of ML and DL Models for Survival Outcome in CVD Prediction

Consistent with studies that have examined both ML and DL models in the context of predicting the survival of cancer patients [76, 79], our study found that DL models surpass ML models in predicting time to CVD occurrence. That is, among the eight studies that compared ML and DL models together, DL models outperformed in seven, whereas ML models excelled in only one study. This is because DL models can improve prediction by (1) enhancing discrimination and calibration, (2) leveraging large datasets effectively, and (3) autonomously learning complex representations for better risk stratification [80].

### XAI Techniques Utilised

All studies included black-box ML models (except Penalised Cox models) and DL models. Black-box models are not explainable unless XAIs are utilised, which means that humans cannot understand how predictions are made [81]. Despite the included studies considering the black-box models, not all studies interpreted their models. Studies that interpreted their models mostly used permutation-based feature importance followed by SHAP value. Using XAIs, studies identified key factors driving predictions and provide transparency in model decision-making. However, feature importance alone cannot ensure a responsible and effective translation of the model into clinical practice.

### SDoH Variables Accounted for in ML and DL Models for Survival Outcome in CVD Prediction

All studies evaluated the standard modifiable cardiovascular risk factors. Biomarkers, imaging features, and variables related to sleep and diet were also considered. However, despite recent studies revealing the major role of SDoH in CVD [25, 82], only a handful of prediction models incorporated a wide range of SDoH variables. Our findings expand on another systematic review aimed at identifying SDoH in ML based CVD prediction models, which also reported that included models did not give much emphasis to SDoH [83]. In this systematic review, most studies considered certain SDoH variables, such as race, education level, and income. However, the use of specific SDoH variables such as race in deploying ML models is controversial [84, 85]. For example, there is a notion that race is a biological construct, rather than a social one, and the race-aware ML model deployment could perpetuate existing biases and discrimination [86, 87]. While we agree that poorly implemented race-conscious models might perpetuate existing biases, including race in ML models’ deployment is helpful for accurate predictions and addressing racial disparities in health outcomes [87, 88]. Additionally, by incorporating race, models can help tailor interventions and allocate resources more effectively to communities in need. Therefore, rather than simply omitting race in the deployment of ML models, it is essential to implement race-aware models with nuanced considerations tailored to the specific context, purpose, and application of the model [88].

### Gender Stratification in CVD Prediction

In 80% of the studies, gender-stratified prediction was overlooked despite gender playing a role in CVD presentation, diagnosis, and survival [89, 90]. Moreover, the role of gender is a critical determinant of CVD as it shapes one’s norms, roles, social relations, and behaviors [91]. Due to the challenges in distinguishing gender and sex from the studies, we used the general term “gender”. Additionally, it is important to acknowledge the following when considering gender versus sex in the deployment of ML models [92, 93]: (1) Viewing gender strictly as a binary biological construct fails to account for the intricate social factors that shape gender identity and expression, (2) Inferring gender solely based on biological sex characteristics can lead to discrimination against transgender and non-binary individuals. Generally, gender-stratified prediction models are beneficial for pinpointing gender-specific predictive factors for tailored and potentially more effective interventions [94]. However, we recommend that gender-stratified prediction models be undertaken after meticulous attention to the representativeness of data, potential biases, and the fundamental factors driving gender disparities in health outcomes.

### Model Validation

Almost all studies internally validated their models. However, a few studies did external validation. Another review also highlighted that most studies did not perform external validation of their ML models [13]. Although external validation is commonly viewed as a critical step in transitioning clinical prediction models from development to implementation, it should not be seen as an automatic green light for model deployment. Moreover, there is no single recommended validation design, external validation is not always essential, and at times, multiple external validations may be required. Generally, the necessity and scope of external validations are contingent upon the intended application of the model and the justification for conducting an external validation study [95].

### Implications for Clinical Practice and Recommendations

AI-based risk prediction models have an increased discrimination ability and accuracy as compared to the conventional multivariable models [96]. However, there are misconceptions that ML requires large amounts of data [97]. Despite ML models often benefiting from large datasets, they can still be effectively applied to smaller health-related datasets as long as the right balance between data quantity and quality is ensured and interpretability is prioritised [97]. It is also imperative to consider the nature of the dataset. For example, when considering longitudinal data with available follow-up time classification-based ML methods should not be used. Right-censoring should be accounted for, since excluding those who lost to follow-up, may result in a biased estimate. ML models for right-censored data have been utilised since 2008 and have recently flourished. Since 2018/19 numerous new models (particularly DL models) for right-censored data with their respective open-source coding packages have become available [66–69]. While it is encouraging that survival ML and DL models are gaining more focus and the development of cutting-edge models is accelerating, their interpretability still poses a challenge. There are open-source XAI methods such as SurvSHAP and SurvLIME for interpreting ML and DL models for right-censored data [27, 28]. However, it is noted that models trained using the PySurvival package, for instance, are not yet supported. Therefore, it is crucial to also focus on their XAI, whether it is model-agnostic or model-specific. In this systematic review, the quality assessment tool PROBAST, typically used for standard prediction models, was employed. However, its application to AI-based prediction models was not direct, leading to the omission or alteration of some signaling questions to evaluate the studies’ quality. Notably, PROBAST + AI tools are currently in development [98, 99], but at this stage, they remain as protocols and should be made available to researchers and decision-makers soon.

Additionally, a standardised measuring tool for most SDoH variables is lacking. SDoH are complex and specific to context and setting, necessitating tailored approaches. Taking these factors into account when measuring SDoH could aid in the creation of effective, context-specific strategies that precisely reflect the impact of SDoH on health outcomes. Inadequately designed SDoH (e.g., race)-sensitive models have the potential to exacerbate existing biases and discrimination within healthcare systems [86, 87]. Consequently, it is imperative to apply nuanced considerations that are specific to the context, purpose, and application of the predictive model. In this systematic review, despite having not differentiated between gender and sex, we found that a common limitation in CVD risk prediction studies is the rarity of gender-specific analysis. Future prediction studies should focus on gender-stratification while incorporating a range of SDoH in the AI prediction models for enhanced prediction and wise decision making.

### Strengths and Limitations

The strengths of this systematic review are its novelty in concisely summarising the ML and DL models utilised for time to CVD outcomes, the applied interpretation techniques, and the assessment of whether SDoH variables or gender-stratification were accounted for. However, despite this systematic review had compared ML and DL in the context of CVD and found that DL is more effective for predicting incident CVD, due to the heterogeneity of studies (e.g., in terms of population, type and number of variables incorporated), we did not do direct comparison through meta-analysis. Additionally, we note that that the most commonly used models also had the best performance. Therefore, our findings may be biased due to the availability of these models for comparison.

## Conclusion

This review identified and compared the different ML and DL models for survival outcomes in CVD prediction. RSF, survival GBM, and Penalised Cox models were the most popular and optimal predicting ML methods. Among DL models, DeepSurv was the most popular and optimal predicting model. Compared to ML models, DL models had better prediction performance. In general, RSF and DeepSurv models were the most popular and better performing models, regardless of the types of variables included (e.g., SDoH) or the population (e.g., community-based, institutionalised). Permutation-based feature importance and SHAP value were the commonly utilised XAI methods for interpretating the AI models. Despite the evidence for SDoH as predictors of CVD and gender-desegregated findings, they were considered by only a few of the included studies. To improve CVD risk prediction and inform clinicians decision-making future studies need to assess SDoH, in addition to the traditional factors and other emerging risk factors. While men and women share many traditional risk factors for CVD, additional gender-specific risk factors and mechanisms are at play. Therefore, it is crucial to consider gender differences when it comes to predicting and managing CVD risks. Moreover, more methodological work is still required to improve ease of interpretability of deep survival learning models, particularly as they have no built-in feature importance methods.

## Electronic Supplementary Material

Below is the link to the electronic supplementary material.

Supplementary Material 1

## Data Availability

Data is provided within the manuscript or supplementary information files.
