# Supplementary material for: A Systematic Review of Artificial Intelligence Models for Time-to-Event Outcome Applied in Cardiovascular Disease Risk Prediction
Source: J Med Syst. 2024 Jul 19;48(1):68. doi: 10.1007/s10916-024-02087-7 (PMC11271333; doi:10.1007/s10916-024-02087-7)
Supplement: Supplementary file 1 — Supplementary Material 1 [file 10916_2024_2087_MOESM1_ESM.pdf]

Journal of Medical Systems

A Systematic Review of Artificial Intelligence Models for Time-to-Event Outcome applied in Cardiovascular Disease Risk Prediction

Supplementary Material

Supplementary Tables

Table S1. Reporting checklist

Table S2. OVID Medline search terms and result

Table S3. Studies excluded at full text screening stage

Table S4. Characteristics of the studies included in the systematic review

Table S5. Characteristics of survival prediction models used for cardiovascular disease risk prediction

Table S6. Patient recruitment year, missing data management, hyperparameter tuning, and software (including libraries/packages) utilised

Table S1. Reporting checklist

Section and Topic	Item #	Checklist item	Location where item is reported*
**TITLE**			
Title	1	Identify the report as a systematic review.	Page 1
**ABSTRACT**			
Abstract	2	See the PRISMA 2020 for Abstracts checklist.	Page 2
**INTRODUCTION**			
Rationale	3	Describe the rationale for the review in the context of existing knowledge.	Page 6 & 7
Objectives	4	Provide an explicit statement of the objective(s) or question(s) the review addresses.	Page 6 & 7
**METHODS**			
Eligibility criteria	5	Specify the inclusion and exclusion criteria for the review and how studies were grouped for the syntheses.	Page 8 & 9, and Table 1
Information sources	6	Specify all databases, registers, websites, organisations, reference lists and other sources searched or consulted to identify studies. Specify the date when each source was last searched or consulted.	Page 9 &10, and Table S5
Search strategy	7	Present the full search strategies for all databases, registers and websites, including any filters and limits used.	9 &10, and Table S2
Selection process	8	Specify the methods used to decide whether a study met the inclusion criteria of the review, including how many reviewers screened each record and each report retrieved, whether they worked independently, and if applicable, details of automation tools used in the process.	Page 10
Data collection process	9	Specify the methods used to collect data from reports, including how many reviewers collected data from each report, whether they worked independently, any processes for obtaining or confirming data from study investigators, and if applicable, details of automation tools used in the process.	Page 10
Data items	10a	List and define all outcomes for which data were sought. Specify whether all results that were compatible with each outcome domain in each study were sought (e.g. for all measures, time points, analyses), and if not, the methods used to decide which results to collect.	Page 10 and Table 1
	10b	List and define all other variables for which data were sought (e.g. participant and intervention characteristics, funding sources). Describe any assumptions made about any missing or unclear information.	Page 10
Study risk of bias assessment	11	Specify the methods used to assess risk of bias in the included studies, including details of the tool(s) used, how many reviewers assessed each study and whether they worked independently, and if applicable, details of automation tools used in the process.	Page 11

Section and Topic	Item #	Checklist item	Location where item is reported*
Effect measures	12	Specify for each outcome the effect measure(s) (e.g. risk ratio, mean difference) used in the synthesis or presentation of results.	Page 10, 11: e.g., prediction performance
Synthesis methods	13a	Describe the processes used to decide which studies were eligible for each synthesis (e.g. tabulating the study intervention characteristics and comparing against the planned groups for each synthesis (item #5)).	Page 10 & 11
	13b	Describe any methods required to prepare the data for presentation or synthesis, such as handling of missing summary statistics, or data conversions.	Page 10 & 11
	13c	Describe any methods used to tabulate or visually display results of individual studies and syntheses.	Page 10 & 11
	13d	Describe any methods used to synthesize results and provide a rationale for the choice(s). If meta-analysis was performed, describe the model(s), method(s) to identify the presence and extent of statistical heterogeneity, and software package(s) used.	
	13e	Describe any methods used to explore possible causes of heterogeneity among study results (e.g. subgroup analysis, meta-regression).	N/A
	13f	Describe any sensitivity analyses conducted to assess robustness of the synthesized results.	N/A
Reporting bias assessment	14	Describe any methods used to assess risk of bias due to missing results in a synthesis (arising from reporting biases).	N/A
Certainty assessment	15	Describe any methods used to assess certainty (or confidence) in the body of evidence for an outcome.	N/A
**RESULTS**			
Study selection	16a	Describe the results of the search and selection process, from the number of records identified in the search to the number of studies included in the review, ideally using a flow diagram.	Page 11 & 12
	16b	Cite studies that might appear to meet the inclusion criteria, but which were excluded, and explain why they were excluded.	Page 11 and Table S3
Study characteristics	17	Cite each included study and present its characteristics.	Page 10 &11, and Table S4
Risk of bias in studies	18	Present assessments of risk of bias for each included study.	Page 23 & 24
Results of individual	19	For all outcomes, present, for each study: (a) summary statistics for each group (where appropriate) and (b) an effect estimate and its precision (e.g. confidence/credible interval), ideally using structured tables or plots.	Table 12 & 13, and Table S4

Section and Topic	Item #	Checklist item	Location where item is reported*
studies			
Results of syntheses	20a	For each synthesis, briefly summarise the characteristics and risk of bias among contributing studies.	Page 12-22, and Table S4
	20b	Present results of all statistical syntheses conducted. If meta-analysis was done, present for each the summary estimate and its precision (e.g. confidence/credible interval) and measures of statistical heterogeneity. If comparing groups, describe the direction of the effect.	N/A
	20c	Present results of all investigations of possible causes of heterogeneity among study results.	N/A
	20d	Present results of all sensitivity analyses conducted to assess the robustness of the synthesized results.	N/A
Reporting biases	21	Present assessments of risk of bias due to missing results (arising from reporting biases) for each synthesis assessed.	N/A
Certainty of evidence	22	Present assessments of certainty (or confidence) in the body of evidence for each outcome assessed.	N/A
**DISCUSSION**			
Discussion	23a	Provide a general interpretation of the results in the context of other evidence.	Page 24-31, Figure 8
	23b	Discuss any limitations of the evidence included in the review.	Page 31-33
	23c	Discuss any limitations of the review processes used.	Page 32 & 33
	23d	Discuss implications of the results for practice, policy, and future research.	Page 31 & 32
**OTHER INFORMATION**			
Registration and protocol	24a	Provide registration information for the review, including register name and registration number, or state that the review was not registered.	Page 8
	24b	Indicate where the review protocol can be accessed, or state that a protocol was not prepared.	Page 8
	24c	Describe and explain any amendments to information provided at registration or in the protocol.	N/A
Support	25	Describe sources of financial or non-financial support for the review, and the role of the funders or sponsors in the review.	Page 31
Competing interests	26	Declare any competing interests of review authors.	Page 34
Availability of	27	Report which of the following are publicly available and where they can be found: template data collection forms;	Page 36

Section and Topic	Item #	Checklist item	Location where item is reported*
data, code and other materials		data extracted from included studies; data used for all analyses; analytic code; any other materials used in the review.	

* Page numbers are assigned during manuscript submission, but they will change once the manuscript is published.

Table S2. OVID Medline search terms and result

No.	Mesh Terms and Keywords	Results from 21 Dec 2023
1	cardiovascular diseases/ or heart diseases/ or cardiomyopathies/ or heart failure/	422,519
2	myocardial ischemia/ or acute coronary syndrome/ or angina pectoris/ or coronary artery disease/ or myocardial infarction/ or peripheral vascular diseases/ or peripheral arterial disease/	343,875
3	cerebrovascular disorders/ or stroke/ or hemorrhagic stroke/ or ischemic stroke/	187,394
4	((cardiovascular or cerebrovascular or cerebral vascular or coronary or heart or cardiac or myocardi*) adj3 (disease* or isch?emi* or infarct* or failure)).mp.	1,142,616
5	(cerebrovascular accident* or ar?hythmia* or arr?ythmia or angina pectoris or unstable angina or stable angina or coronary syndrome or stroke or adverse cardiac event or major adverse cardiovascular event* or heart attack* or cardiovascular mortality or cardiovascular death or out-of-hospital cardiac arrest or cardiomyopath* or (peripheral adj2 disease*)).mp.	698,884
6	or/1-5	1,587,346
7	algorithms/ or artificial intelligence/ or machine learning/ or deep learning/ or supervised machine learning/	369,070
8	(Artificial intelligence or machine learning or deep learning or random survival forest or Extra Survival Trees or survival ensembles or boosting or survival support vector machine or Multi-Task Logistic Regression or DeepSurv or Non Linear Cox proportional hazard model* or Cox-time or CoxTime or Cox-CC or CoxCC or probability mass function or Nnet-survival or DeepHit or DeepHitSingle or Piecewise Constant Hazard model* or Discrete-Time Model* or Continuous-Time Model* or Neural network* or deep neural survival network*).mp.	192,170
9	or/7-8	450,589
10	Forecasting/ or Risk Assessment/ or Prognosis/	959,838
11	(predict* or risk or progno* or detect* or identif* or forecasting).mp.	8,898,601
12	or/10-11	8,898,601
13	survival analysis/	145,869
14	(time to event or censor* or survival).mp.	1,373,208
15	or/13-14	1,373,208
16	6 and 9 and 12 and 15	866

Table S3. Studies excluded at full text screening stage

No.	Title	Authors	Year of publication	Journal	DOI	Notes
1.	Lifetime vs 10-year Cardiovascular Disease Prediction in Young Adults Using Statistical Machine Learning and Deep Learning: The CARDIA Study	Ambale-Venkatesh, B.; Nguyen, H. T.; Reis, J. P.; Wu, C. O.; Carr, J. J.; Nwabuo, C.; Gidding, S. S.; Guallar, E.; Lima, J. A. C.	2022	medRxiv	https://dx.doi.org/10.1101/2022.09.22.22280254	Unpublished work/preprint
2.	Using machine learning methods to identify predictors of incident myocardial infarction in the women's health initiative cohort	Avram, R.; Tison, G.; Nah, G.; Howard, B. V.; Olgin, J.; Parikh, N. I.	2018	Circulation		Conference abstract
3.	Machine learning for time-to-event analysis in patients with suspected coronary artery disease: increased long-term prognostic value of coronary CT angiography-derived measures and clinical parameters	Bauer, M. J.; Nano, N.; Adolf, R.; Will, A.; Hendrich, E.; Martinoff, S.; Hadamitzky, M.	2022	Insights into Imaging	https://dx.doi.org/10.1186/s13244-022-01337-x	Conference abstract
4.	A novel risk prediction model of atrial fibrillation: The multi-ethnic study of atherosclerosis (MESA)	Bundy, J. D.; Heckbert, S. R.; Chen, L. Y.; Lloyd-Jones, D. M.; Greenland, P.	2018	Circulation		Wrong outcome
5.	Associations of Inflammation with Risk of Cardiovascular and All-Cause Mortality in Adults with Hypertension: An Inflammatory Prognostic Scoring System	Cheang, I.; Zhu, X.; Lu, X.; Yue, X.; Tang, Y.; Gao, R.; Liao, S.; Yao, W.; Zhou, Y.; Zhang, H.; Yiu, K. H.; Li, X.	2022	Journal of Inflammation Research	https://dx.doi.org/10.2147/JIR.S384977	Not intended for prediction
6.	Machine learning can predict survival of patients with heart failure from serum creatinine and ejection fraction alone	Chicco, Davide; Jurman, Giuseppe	2020	BMC Medical Informatics and Decision Making	https://dx.doi.org/10.1186/s12911-020-1023-5	Wrong outcome
7.	Machine learning to predict the long-term risk of myocardial infarction and cardiac death based on clinical risk, coronary calcium and epicardial adipose tissue: A prospective study	Commandeur, F. C.; Slomka, P. J.; Goeller, M.; Chen, X.; Cadet, S.; Razipour, A.; Gransar, H.; Cantu, S.; Miller, R.; Rozanski, A.; Achenbaclh, S.; Tamarappoo, B.; Berman, D.; Dey, D.	2019	European Heart Journal	https://dx.doi.org/10.1093/eurheartj/ehz747.0002	Conference abstract
8.	Atherosclerotic cardiovascular events prediction using machine learning models: Results from action to control cardiovascular risk in diabetes trial	Fan, W.	2020	Circulation	https://dx.doi.org/10.1161/circ.141.suppl-1.MP58	Not a ML technique for survival outcomes
9.	Machine learning-based prediction of 1-year mortality for acute coronary syndrome	Hadanny, Amir; Shouval, Roni; Wu, Jianhua; Gale, Chris P.; Unger, Ron; Zahger, Doron; Gottlieb, Shmuel; Matetzky, Shlomi; Goldenberg, Ilan; Beigel, Roy; Iakobishvili, Zaza	2022	Journal of cardiology	https://dx.doi.org/10.1016/j.jjcc.2021.11.006	Wrong outcome
10.	Cardiovascular risk stratification through deep neural survival networks - the multi-ethnic study of atherosclerosis (MESA)	Hathaway, Q.; Yanamala, N.; Budoff, M.; Sengupta, P.; Zeby, I.	2021	Journal of the American College of Cardiology	https://dx.doi.org/10.1016/S0735-1097%2821%2901920-3	Conference abstract
11.	PMHnet-alpha: Development and validation of a neural network based discrete-time survival model for mortality prediction in ischemic heart disease	Holm, P.; Haue, A. D.; Westergaard, D.; Banasik, K.; Koeber, L.; Brunak, S.; Bundgaard, H.	2022	European Respiratory Journal	https://dx.doi.org/10.1093/eurheartj/ehac544.2785	Conference abstract
12.	Identifying important risk factors for survival in patient with systolic heart failure using random survival forests	Hsich, E.; Gorodeski, E. Z.; Blackstone, E. H.; Ishwaran, H.; Lauer, M. S.	2011	Circulation: Cardiovascular Quality and Outcomes	https://dx.doi.org/10.1161/CIRCOUTCOMES.110.939371	Wrong outcome
13.	Predictors of Major Adverse Cardiovascular Events Among Type 2 Diabetes Mellitus Patients: A Machine Learning Time-to-Event Analysis	Icten, Z.; Friedman, M.; Menzin, J.	2022	Value in Health	https://dx.doi.org/10.1016/j.jval.2022.04.032	Conference abstract

14.	Prognostic Implications of Coronary CT Angiography: 12-Year Follow-Up of 6892 Patients	Johnson, Kevin M.; Dowe, David A.	2020	American journal of roentgenology	https://dx.doi.org/10.2214/AJR.19.22578	Not a ML technique for survival outcomes
15.	Deep learning survival analysis enhances the value of hybrid PET/CT for long-term cardiovascular event prediction	Juarez-Orozco, L. E.; Benjamins, J. W.; Maaniitty, T.; Saraste, A.; Van Der Harst, P.; Knuuti, J.	2019	EUROPEAN HEART JOURNAL	https://dx.doi.org/10.1093/eurheartj/ehz748.0177	Conference abstract
16.	Outcome predictions using machine learning in atrial fibrillation-related stroke	Jung, J. M.; Jeon, E. T.	2021	Circulation	https://dx.doi.org/10.1161/circ.144.suppl-1.11932	Conference abstract
17.	Comparing data-driven 10-year cardiovascular disease risk prediction using boosted regression trees to a mainstreamed risk prediction algorithm	Ka Chun Tsang, K. C.; Norberg, M.; Naslund, U.; Weinehall, L.; Carlberg, B.; Wennberg, P.; Ng, N.; Lindahl, B.; Rocklov, J.	2017	European Journal of Preventive Cardiology	-	Conference abstract
18.	A simple risk score to predict mortality for patients with heart failure with preserved ejection fraction-a report from the chart-2 study	Kasahara, S.; Sakata, Y.; Nochioka, K.; Abe, R.; Oikawa, T.; Sato, M.; Aoyanagi, H.; Shiroto, T.; Takahashi, J.; Miyata, S.; Shimokawa, H.	2017	Circulation	-	Wrong outcome
19.	Development of a simple risk score to predict mortality of patients with chronic heart failure with preserved ejection fraction	Kasahara, S.; Sakata, Y.; Nochioka, K.; Abe, R.; Oikawa, T.; Sato, M.; Shiroto, T.; Takahashi, J.; Miyata, S.; Shimokawa, H.	2017	Journal of Cardiac Failure	https://dx.doi.org/10.1016/j.cardfail.2017.08.277	Not intended for prediction
20.	Identification of Risk Factors for Mortality after Myocardial Infarction Using Machine Learning Methods	Kashirina, I. L.; Firyulina, M. A.; Bondarenko, Y. V.; Desyatirikova, E. N.; Efimova, O. E.; Chernenkaya, L. V.	2021	International Conference on Soft Computing and Measurements (SCM)	https://doi.org/10.1109/SCM52931.2021.9507190	Wrong outcome
21.	Use of machine learning to predict drivers of incident heart failure in patients with type 2 diabetes mellitus	Kaur, N.; Pellicori, P.; Deligianni, F.; Clelland, J. G. F.	2023	Heart	https://dx.doi.org/10.1136/heartjnl-2023-BCS.140	Conference abstract
22.	Machine learning-based approach for predicting post-treatment survival for patients with coronary artery disease	Khalafbeigi, A.; Kalmady, S.; Bainey, K.; Welsh, R.; Kaul, P.; Greiner, R.	2023	Canadian Journal of Cardiology	https://dx.doi.org/10.1016/j.cjca.2023.06.328	Conference abstract
23.	An integrated machine learning approach to stroke prediction	Khosla, A.; Cao, Y.; Lin, C. C. Y.; Chiu, H. K.; Hu, J.; Lee, H.	2010		https://dx.doi.org/10.1145/1835804.1835830	Conference abstract
24.	Predicting survival in heart failure: a risk score based on machine-learning and change point algorithm	Kim, Wonse; Park, Jin Joo; Lee, Hae-Young; Kim, Kye Hun; Yoo, Byung-Su; Kang, Seok-Min; Baek, Sang Hong; Jeon, Eun-Seok; Kim, Jae-Joong; Cho, Myeong-Chan; Chae, Shung Chull; Oh, Byung-Hee; Kook, Woong; Choi, Dong-Ju	2021	Clinical research in cardiology : official journal of the German Cardiac Society	https://dx.doi.org/10.1007/s00392-021-01870-7	Wrong outcome
25.	Use of neural networks in predicting the risk of coronary artery disease	Lapuerta, P.; Azen, S. P.; LaBree, L.	1995	Computers and biomedical research	-	Not a ML technique for survival outcomes

26.	Machine learning-based models to predict one-year mortality among Chinese older patients with coronary artery disease combined with impaired glucose tolerance or diabetes mellitus	Li, Yan; Guan, Lixun; Ning, Chaoxue; Zhang, Pei; Zhao, Yali; Liu, Qiong; Ping, Ping; Fu, Shihui	2023	Cardiovascular Diabetology	https://dx.doi.org/10.1186/s12933-023-01854-z	Wrong outcome
27.	Cardiovascular risk prediction using machine learning in a large Japanese cohort	Matheson, M. B.; Kato, Y.; Baba, S.; Cox, C.; Lima, J. A.; Venkatesh, B. A.	2021	Circulation	https://dx.doi.org/10.1161/circ.143.suppl_1.011	Conference abstract
28.	Predictive modeling of hospital mortality for patients with heart failure by using an improved random survival forest	Miao, F.; Cai, Y. P.; Zhang, Y. X.; Fan, X. M.; Li, Y.	2018	IEEE Access	https://dx.doi.org/10.1109/ACCESS.2018.2789898	Wrong outcome
29.	A machine learning-based model to predict the 15-year risk for cardiovascular disease in a cohort of people living with HIV	Muccini, C.; Masci, C.; Corso, F.; Galli, L.; Poli, A.; Ranzenigo, M.; Monardo, R.; Paganoni, A. M.; Castagna, A.; Leva, F.	2021	HIV Medicine	https://dx.doi.org/10.1111/hiv.13183	Conference abstract
30.	Risk factor structure of heart failure in patients with cancer after treatment with anticancer agents' assessment by big data from a Japanese electronic health record	Nohara, Shoichiro; Ishii, Kazuo; Shibata, Tatsuhiro; Obara, Hitoshi; Miyamoto, Takanobu; Ueno, Takafumi; Kakuma, Tatsuyuki; Fukumoto, Yoshihiro	2023	Heart and Vessels	https://dx.doi.org/10.1007/s00380-023-02238-9	Not intended for prediction
31.	Cardiovascular Disease Risk Prediction by Random Survival Forest: The Korean National Health Insurance Service-National Health Screening Cohort	Park, S.; Ratcliffe, S.; Bowles, K.; Ulrich, C. M.	2023	Circulation	https://dx.doi.org/10.1161/circ.148.suppl_1.15002	Conference abstract
32.	Predictors of hospitalization or death due to heart failure in diabetic patients by gender in the accord trial using random survival forests	Patel, T.; Shamsuzzaman, M.; Wu, C.; Almario, E. N.; Tesfaldet, B.; Fleg, J.; Csako, G.; Gandotra, C.; Sopko, G.; Sviglin, H.; Coady, S.; Burkhart, K.; Calis, K.; Cooper, L.; Amin, N.; Banerjee, A.; Farooque, N.; Taylor, A.; Gupta, S.; Dodge, A.; Dandi, G.; Hoque, L.; Fennessy, M.; Raman, S.; Kirby, R.; Chen, J.; Yan, Y.; Liu, L.; Leifer, E.; Chang, H.; Cure, C.; Desvigne-Nickens, P.; Szarfman, A.; Domanski, M.; Pucino, F.; Rosenberg, Y.; Hasan, A.	2017	Circulation	-	Conference abstract
33.	Machine-learning score using stress CMR and CCTA for prediction of cardiovascular events in patients with obstructive CAD	Pezel, T.; Garot, P.; Toupin, S.; Hamzi, K.; Hovasse, T.; Lefevre, T.; Unterseeh, T.; Sanguineti, F.; Goncalves, T.; Dillinger, J. G.; Bousson, V.; Henry, P.; Garot, J.	2023	Archives of Cardiovascular Diseases Supplements	https://dx.doi.org/10.1016/j.acvdsp.2023.04.027	Conference abstract
34.	Prediction of Major Adverse Cardiac Events after Myocardial Perfusion Imaging using multi-task deep neural network and time-to-event data	Pieszko, K.; Singh, A.; Killekar, A.; Otaki, Y.; Sharir, T.; Einstein, A. J.; Fish, M. B.; Ruddy, T. D.; Kaufmann, P.; Sinusas, A. J.; Miller, E. J.; Bateman, T. M.; Dorbala, S.; Di Carli, M.; Dey, D.; Liang, J.; Berman, D. S.; Slomka, P. J.	2021	European Journal of Nuclear Medicine and Molecular Imaging	https://dx.doi.org/10.1007/s00259-021-05547-1	Conference abstract
35.	Convolutional multi-task deep neural network precisely predicts time-dependent survival of majoradverse cardiac eventsafter myocardial perfusion imaging	Pieszko, K.; Singh, A.; Otaki, Y.; Sharir, T.; Einstein, A. J.; Fish, M. B.; Ruddy, T. D.; Kaufmann, P. A.; Sinusas, A. J.; Miller, E. J.; Bateman, T. M.; Dorbala, S.; Di Carli, M.; Dey, D.; Liang, J. X.; Berman, D. S.; Slomka, P. J.	2021	Journal of Nuclear Cardiology	https://dx.doi.org/10.1007/s12350-021-02760-1	Conference abstract
36.	An Explainable Transformer-Based Deep Learning Model for the Prediction of Incident Heart Failure	Rao, S.; Li, Y.; Ramakrishnan, R.; Hassaine, A.; Canoy, D.; Cleland, J.; Lukasiewicz, T.; Salimi-Khorshidi, G.; Rahimi, K.	2022	IEEE Journal of Biomedical and Health Informatics	https://dx.doi.org/10.1109/JBHI.2022.3148820	Not a ML technique for survival outcomes

37.	Predictive performance of machine learning models for detection of incident heart failure using multicentre data	Sabovcik, F.; Ntalianis, E.; Cauwenberghs, N.; Kuznetsova, T.	2022	Journal of Hypertension	https://dx.doi.org/10.1097/01.hjh.0000835320.43093.76	Conference abstract
38.	A novel risk prediction score for incident heart failure among patients with diabetes	Segar, M. W.; Patel, K. V.; Berry, J. D.; Pandey, A.	2019	Circulation	https://dx.doi.org/10.1161/circ.139.suppl_1.P378	Conference abstract
39.	Model Complexity and Explainability in Prediction for Coronary Artery Disease in the UK Biobank	Sharapova, N.; Maxwell, J. M.; Hagenaars, S. P.; Russell, R. A.; Ibrahim, Z. M.; Lewis, C. M.	2022	Genetic Epidemiology	https://dx.doi.org/10.1002/gepi.22503	Conference abstract
40.	Predicting ischemic stroke and all-cause mortality risk in patients with heart failure with reduced ejection fraction and sinus rhythm: A secondary analysis of the warcef trial	Sharma, R.; Krumholz, H. M.; Sheth, K. N.; Faridi, K.; Kamel, H.; Merkler, A. E.	2022	Stroke	https://dx.doi.org/10.1161/str.53.suppl_1.TP188	Conference abstract
41.	Predicting major adverse cardiac events with cox neural networks: results from the refine spect registry	Slomka, P.; Betancur, J.; Otaki, Y.; Commandeur, F.; Sharir, T.; Einstein, A.; Fish, M.; Ruddy, T.; Kaufmann, P. A.; Sinusas, A.; Miller, E.; Bateman, T.; Dorbala, S.; Di Carli, M.; Diniz, M.; Germano, G.; Dey, D.; Cooper, L.; Berman, D.	2019	Journal of the American College of Cardiology	https://dx.doi.org/10.1016/S0735-1097%2819%2932038-8	Conference abstract
42.	ASCVD Risk Score vs Machine Learning-Based Algorithm in the Prediction of ASCVD Events in Women With Breast Cancer	Stabellini, N.; Blumenthal, R. S.; Bittencourt, M. S.; Whelton, S. P.; Leong, D.; Moore, J.; Cullen, J.; Nain, P.; Shanahan, J.; Dent, S. F.; Montero, A.; Guha, A.	2023	Circulation	https://dx.doi.org/10.1161/circ.148.suppl_1.14810	Not intended for prediction
43.	Prognostication of Incidence and Severity of Ischemic Stroke in Hot Dry Climate From Environmental and Non-Environmental Predictors	Statsenko, Y.; Habuza, T.; Fursa, E.; Ponomareva, A.; Almansoori, T. M.; Zahmi, F. A.; Gorkom, K. N. V.; Laver, V.; Talako, T.; Szolics, M.; Dehdashtian, A.; Koteesh, J. A.; Ljubisavljevic, M.	2022	IEEE Access	https://dx.doi.org/10.1109/ACCESS.2022.3175302	Not a ML technique for survival outcomes
44.	Use of Machine Learning and Prediction Tools to Assess Cardiovascular Disease Risk in Obstructive Sleep Apnea	Suarez-Farinas, M.; Cohen, O.; Al-Taie, Z.; Khan, S.; Nadkarni, G.; Barbe, F.; Sanchez-de-la-Torre, M.; Shah, N. A.	2023	American Journal of Respiratory and Critical Care Medicine	https://dx.doi.org/10.1164/ajrccm-conference.2023.C98	Conference abstract
45.	Prediction of 30-day mortality in heart failure patients with hypoxic hepatitis: Development and external validation of an interpretable machine learning model	Sun, R.; Wang, X.; Jiang, H.; Yan, Y.; Dong, Y.; Yan, W.; Luo, X.; Miu, H.; Qi, L.; Huang, Z.	2022	Frontiers in Cardiovascular Medicine	https://dx.doi.org/10.3389/fcvm.2022.1035675	Wrong outcome
46.	Identifying novel predictors for incident heart failure using statistical learning techniques in the women's health initiative (WHI) cohort	Tison, G. H.; Nah, G.; Olgin, J. E.; Vittinghoff, E.; Howard, B. V.; Foraker, R.; Allison, M. A.; Casanova, R. L.; Blair, R. H.; Breathett, K. K.; Klein, L.; Parikh, N. I.	2016	Circulation	-	Not intended for prediction
47.	Cardiovascular Risk Assessment Using Artificial Intelligence-Enabled Event Adjudication and Hematologic Predictors	Truslow, James G.; Goto, Shinichi; Homilius, Max; Mow, Christopher; Higgins, John M.; MacRae, Calum A.; Deo, Rahul C.	2022	Circulation. Cardiovascular quality and outcomes	https://dx.doi.org/10.1016/S0735-1097%2819%2931298-7	Not intended for prediction
48.	Machine Learning Models to Predict Development of CKD and/or HF in Early Stages of Type 2 Diabetes Patients	Tsubota, H.; Yajima, T.; Kanda, E.; Kanemata, S.; Suzuki, A.; Shirakawa, K.; Makino, M.	2022	Circulation	https://dx.doi.org/10.1161/circ.146.suppl_1.11780	Wrong outcome
49.	Machine learning to predict cardiometabolic outcomes in people living with overweight and obesity	Turchin, A.; Morrison, F.; Shubina, M.; Shinde, S.; Ahmad, N.; Kan, H.	2021	Obesity	https://dx.doi.org/10.1002/oby.23328	Conference abstract

50.	Interpretable prediction of 3-year all-cause mortality in patients with heart failure caused by coronary heart disease based on machine learning and SHAP	Wang, K.; Tian, J.; Zheng, C.; Yang, H.; Ren, J.; Liu, Y.; Han, Q.; Zhang, Y.	2021	Computers in Biology and Medicine	https://dx.doi.org/10.1016/j.compbiomed.2021.104813	Wrong outcome
51.	Improving the Prediction of Death from Cardiovascular Causes with Multiple Risk Markers	Wang, X.; Bakulski, K. M.; Fansler, S.; Mukherjee, B.; Park, S. K.	2023	medRxiv	https://dx.doi.org/10.1101/2023.01.21.23284863	Unpublished research work
52.	Risk stratification for mortality in cardiovascular disease survivors: A survival conditional inference tree analysis	Wu, Zhijun; Huang, Zhe; Wu, Yuntao; Jin, Yao; Wang, Yanxiu; Zhao, Haiyan; Chen, Shuohua; Wu, Shouling; Gao, Xiang	2021	Nutrition, metabolism, and cardiovascular diseases	https://dx.doi.org/10.1016/j.numecd.2020.09.029	Wrong outcome
53.	Gender and Age Specific Baseline Predictors of MACE in PEACE Trial Identified by Machine Learning	Xin, V.; Hayashi, S.; Husain, A.; Hasan, A. A.; Dey, A.; Banerjee, A.; Atkinson, I.; Dandi, G.; Qureshi, K.; Lewis, N.; Mahmood, N.; Hasan, N.; Haq, N.; Gani, N.; Mallick, Z.; Rosenberg, Y. D.	2020	Circulation	https://dx.doi.org/10.1161/circ.142.suppl_3.16998	Not intended for prediction
54.	Machine learning to predict the long-term risk of myocardial infarction and cardiac death based on clinical risk, coronary calcium, and epicardial adipose tissue: a prospective study	Commandeur, F.; Slomka, P. J.; Goeller, M.; Chen, X.; Cadet, S.; Razipour, A.; McElhinney, P.; Gransar, H.; Cantu, S.; Miller, R. J. H.; Rozanski, A.; Achenbach, S.; Tamarappoo, B. K.; Berman, D. S.; Dey, D.	2020	Cardiovascular Research	https://dx.doi.org/10.1093/cvr/cvz321	Not a ML technique for survival outcomes
55.	Predicting Cardiovascular Disease Mortality: Leveraging Machine Learning for Comprehensive Assessment of Health and Nutrition Variables	Martin-Morales, A.; Yamamoto, M.; Inoue, M.; Vu, T.; Dawadi, R.; Araki, M.	2023	Nutrients	https://dx.doi.org/10.3390/nu15183937	Not a ML technique for survival outcomes
56.	Development and Validation of Machine Learning-Based Race-Specific Models to Predict 10-Year Risk of Heart Failure A Multicohort Analysis	Segar, M. W.; Jaeger, B. C.; Patel, K. V.; Nambi, V.; Ndumele, C. E.; Correa, A.; Butler, J.; Chandra, A.; Ayers, C.; Rao, S.; Lewis, A. A.; Raffield, L. M.; Rodriguez, C. J.; Michos, E. D.; Ballantyne, C. M.; Hall, M. E.; Mentz, R. J.; de Lemos, J. A.; Pandey, A.	2021	Circulation	https://dx.doi.org/10.1161/circulationaha.120.053134	Duplicate
57.	Machine Learning-Based Models Incorporating Social Determinants of Health vs Traditional Models for Predicting In-Hospital Mortality in Patients With Heart Failure	Segar, M. W.; Hall, J. L.; Jhund, P. S.; Powell-Wiley, T. M.; Morris, A. A.; Kao, D.; Fonarow, G. C.; Hernandez, R.; Ibrahim, N. E.; Rutan, C.; Navar, A. M.; Stevens, L. M.; Pandey, A.	2022	Jama Cardiology	https://dx.doi.org/10.1001/jamacardio.2022.1900	Wrong outcome
58.	Comment on Segar et al. Machine Learning to Predict the Risk of Incident Heart Failure Hospitalization Among Patients With Diabetes: The WATCH-DM Risk Score. Diabetes Care 2019;42:2298-2306	Shao, H.; Shi, L. Z.; Fonseca, V.	2020	Diabetes Care	https://dx.doi.org/10.2337/dc19-1891	Letter
59.	Using machine learning to predict adverse events in acute coronary syndrome: A retrospective study	Song, L.; Li, Y.; Nie, S. S.; Feng, Z. Y.; Liu, Y. X.; Ding, F. F.; Gong, L. Y.; Liu, L. M.; Yang, G. P.	2023	Clinical Cardiology	https://dx.doi.org/10.1002/clc.24127	Not a ML technique for survival outcomes
60.	Analyzing and predicting the risk of death in stroke patients using machine learning	Zhu, E. Z.; Chen, Z. H.; Ai, P.; Wang, J. Y.; Zhu, M.; Xu, Z. Q.; Liu, J.; Ai, Z. S.	2023	Frontiers in Neurology	https://dx.doi.org/10.3389/fneur.2023.1096153	Wrong outcome
61.	Deep phenotyping and prediction of long-term heart failure by machine learning	Zhuang, X.; Sun, X.; Zhong, X.; Zhou, H.; Zhang, S.; Liao, X.	2019	Journal of the American College of Cardiology	https://dx.doi.org/10.1016/S0735-1097%2819%2931298-7	Duplicate

Table S4. Characteristics of the studies included in the systematic review.

Author and year	Journal	Population	Study region (country)	Setting	Length of follow up	End point (predicted outcome)	Sample size	Age	Gender (% women)	Event (%)	End point definition
Ambale-Venkatesh 2017 (1)	Circulation research	Participants free of clinical CVD at baseline. (MESA cohort)	United States	Community	Median (IQR) in year: 11.2 (10.6–11.7)	CVD, CHD Stroke, and HF	6,814	Mean (SD) age in year: 62.15 (10.23)	53.0%	CVD: 10.42%; CHD: 7.31%; Stroke: 2.94%; and HF: 3.80%	CVD represented a composite of CVD death, stroke, and CHD. CHD included any of MI, resuscitated cardiac arrest, definite angina, probable angina followed by revascularization, and CHD death. Stroke was defined as rapid onset of a documented focal neurological deficit (vascular causes) lasting 24 hours or until death, or if <24 hours, when there was a clinically relevant brain lesion. HF included symptomatic HF diagnosed by a physician and patient receiving medical treatment for HF, in addition to (1) pulmonary edema/ congestion, and (2) dilated ventricle or poor left ventricular function, or evidence of left ventricular diastolic dysfunction.
Barbieri 2022 (2)	International Journal of Epidemiology	Participants with no history of CVD or heart failure (administrative data)	New Zealand	Community	Mean: 4.8 years in women and 4.7 years in men	Fatal or non-fatal CVD	2,164, 872	Mean age (SD) in year: 49.0 (11.8) in both men and women	52.7%	Fatal and non-fatal CVD: 2.1% for women and 3.7% for men	A CVD event was defined as hospitalisation with a discharge diagnosis code consistent with CVD or heart failure. A death was classified as a CVD death if the underlying cause is either cardiac arrest, ischemic heart disease, coronary procedure, stroke, peripheral vascular disease, or congestive heart failure.
Bauer 2023 (3)	Radiology: Cardiothoracic Imaging	Patients with suspected CAD who underwent Coronary CT Angiography (administrative data)	NR	Institution	Median (IQR) in year: 7.3 (4.5–9.8)	MACE	5,457	Mean age (SD) in year: 61 (11)	33.2%	5.57%	MACE was defined as the composite of all-cause death, MI, unstable angina, or late revascularization (>90 days after index scan).
Blanchard 2022 (4)	IEEE Access	Participants without a history of MACE (IRSR-PLSC cohort)	France	community	Median (IQR) in year: 6.0 (3.9-8.7)	MACE	5,506	Median age (IQR) in year: 59 (49–69)	39.4%	11.13%	MACE was defined as the first hospitalization due to MI, stroke, exacerbation of congestive heart failure, revascularization procedure (percutaneous coronary intervention, coronary artery

											bypass graft surgery), or all-cause death.
Brester 2023 (5)	Biostatistics and Epidemiology	Middle-aged men, 42–60 years old (KIHD risk factor study)	Finland	Community	Maximum: 30-year	CVD mortality	2,682	Range in year: 42-60	0%	NR	CVD mortality referring to codes I00–I99 of the 10th International Classification of Diseases (ICD-10).
Chhoa 2023 (6)	Scientific reports	Aged over 18 years were diagnosed with Cerebral autosomal dominant arteriopathy with subcortical infarcts and leukoencephalopathy (administrative data)	France	Institution	Maximum: 5-year	Stroke or death	422	NR	NR	34.12%	Time to either ischemic and hemorrhagic strokes or death, whichever occurred first.
Chun 2021 (7)	Journal of the American Medical Informatics Association	Participants without disability aged 35–74 years with no prior history of stroke or TIA at baseline (CKB cohort)	China	Community	Maximum: 9-year	Stroke	503,842	Mean (SD) age in year: 51.9 (10.6)	59%	Men: 9.5%; Women: 7.9%.	All fatal and non-fatal stroke cases based on the International Classification of Diseases 10th revision (ICD-10).
Deng 2023 (8)	BMC medical research methodology	Participants aged 40-79 years who are free of a previous history of MI, stroke, congestive heart failure, or atrial fibrillation. (Lifetime risk pooling project)	United States	Community	Mean (SD) in year: 10.50 (3.02)	ASCVD	23,216	Mean (SD) in year: 57.8 (9.6)	56.93%	16%	ASCVD was defined as nonfatal MI or CHD death, or fatal or nonfatal stroke
Duan 2024 (9)	Ecotoxicology and Environmental Safety	Adults (aged ≥18 years) (NHANES)	United States	community	Mean (SD) in year: 7.8 (0.6)	CVD mortality	1,602	Mean (SD) in year: 46.33 (18.39)	48.94%	2.12%	CVD mortality (I00-I09, I11, I13, I20-I51)
Farhadian 2021 (10)	BMC Cardiovascular Disorders	Adult patients undergoing coronary angioplasty (administrative data)	Iran	Institution	Mean in year: 8.05	MACE	220	Mean (SD) age in year: 60.00 (11.09)	31.4%	43.7%	MACE defined as a composite of All-cause death, coronary artery bypass graft surgery, stroke, and repeat revascularization.
Feng 2022 (11)	BMC medical research methodology	Newly diagnosed hypertensive patients aged 18 to 99 years (administrative data)	Canada	Institution	Median (IQR) in year: 3.5 (2.2-4.8)	Hospitalisation attributable to CVD	259,873	Mean (SD) in year: 56.6 (14.0). Median (IQR) in year: 56.1 (47.2-65.8)	47.0%	4.56%	Hospitalisation attributable to as major adverse events; a composite of MI, resuscitated cardiac arrest, congestive heart failure, coronary revascularization, or all-cause death.
Gandin 2023 (12)	PLoS ONE	Adult patients with diabetes Cardiovascular Observatory of Trieste (Italy)	Italy	Institution	Median in year: 5.4	HF	10,614	Mean (SD) in year: 72 (11)	42.0%	17.3%	HF identified as the first between the following events: diagnosis of HF during hospitalization (ICD-9 codes:

											39891, 40201, 40211, 40291, 40401, 40403, 40411, 40413, 40491, 40493, 4280–4284, 4289) and diagnosis of HF based at out-of-hospital clinical examination according to ESC criteria: typical symptoms (breathlessness, ankle swelling and fatigue) and/or signs (elevated jugular venous pressure, pulmonary crackles, and peripheral oedema) in presence of a structural and/or functional cardiac abnormality
Gao 2023 (13)	European radiology	Adult patients diagnosed with HF with reduced ejection fraction (≤40%) according to the ACC/AHA guidelines (administrative data)	China	Institution	Median (IQR) in year: 2.85 (0.58-3.39)	MACE	329	Mean (SD) in year: 54.0 (14.0)	22.8%	18.8%	MACE includes cardiovascular death, rehospitalization because of cardiac dysfunction, and cardiac transplantation.
Garcia-Carretero 2019 (14)	Medical and Biological Engineering and Computing	Adult patients with Hypertension without a history of CVD (administrative data)	Spain	Institution	Median in year: 3.5	MACE	1,471	Mean (SD) in years: 58.1 (12.8)	49.8%	22.43%	MACE was a composite of incident, non-fatal CHD (acute MI), HF, stroke, and cardiovascular death.
Hathaway 2021 (15)	Computers in Biology and Medicine	Multiethnic participants aged 45–84 years old (MESA cohort)	United States	Community	Maximum: 16-year	MACE	6,814	Age range in year: 45–84 (mean age in year: 62.52 SD: 10.19)	53.29%	28.4%	MACE was defined as a composite of MI, resuscitated cardiac arrest, congestive heart failure, coronary revascularization, or all-cause mortality.
Jain 2021 (16)	Journal of Cardiothoracic and Vascular Anesthesia	Adult patients undergoing liver transplantation (administrative data)	United States	Institution	Mean (SD) in year: 4.4 (3.3)	CVD mortality	1,459	Median (IQR) in year: 58 (51-64)	66.0%	3.2%	CVD mortality was defined as death attributable to MI, HF, cardiac arrest, or stroke
Kim 2023 (17)	Journal of neurology, neurosurgery, and psychiatry	Adult patients with acute ischaemic stroke admitted to a stroke Centre (administrative data)	Republic of Korea	Institution	Maximum: 1 year	MACE	8,590	Mean age in year: 71.0	42.49%	13.97%	MACE was defined as a composite of recurrent stroke, acute MI or death). Recurrent stroke was defined as the sudden development of a new stroke or worsening of an existing neurologic deficit after AIS, with evidence of attributable new stroke lesions on brain imaging using CT or MRI.

Lin 2023 (18)	International Journal of Environmental Research and Public health	Adult patients who had acute ischemic stroke and admitted to a hospital (administrative data)	China (Taiwan)	Institution	Median (IQR) in year: 3.2 (1.4-5.6)	CVD mortality	21,463	Mean (SD) in year: 67.33 (12.93)	38.15%	The overall incidence rate: 33.7/1000 person-years	CVD were identified using the International Classification of Diseases, 10th revision, codes (ICD-10-CM codes I00–I99).
Mauger 2023 (19)	Radiology	Adults with diverse race and ethnicity with no clinically apparent CVD (MESA cohort)	United States	Community	Median: 8.5 years)	CVD, HF, and CHD	4,618	Mean (SD) in year: 60.6 (9.9)	55.0%	CVD: 10%; HF:3%; CHD: 7%	CVD events included stroke, CHD, atherosclerotic death, stroke death, and CVD related death. Criteria for CHD included MI, resuscitated cardiac arrest, definite and probable angina, and CHD death. HF included symptomatic HF diagnosed by a physician and treatment, while definite HF also required evidence of one or more other criteria (including pulmonary edema and/ or congestion at chest radiography, a dilated ventricle or poor left ventricular function at echocardiography or ventriculography, or evidence of left ventricular diastolic dysfunction).
Moreno-Sánchez 2023 (20)	Frontiers in Cardiovascular Medicine	Adult patients who suffered an HF episode (administrative data)	Pakistan	Institution	Mean (SD) in month: 4.3 (2.6)	HF mortality	299	Mean (SD) in year: 60.83 (11.89)	35.12%	32.12%	Death due to HF
Morris 2023 (21)	PLoS ONE	Adult population with no history of CVD at baseline (Jackson Heart Study)	United States	Community	Maximum: 10-year	CVD	3,980	Mean (SD) in year: 53.8 (12)	64.0%	9.6%	CVD events included CHD (i.e., definite or probable MI, definite fatal CHD, cardiac procedures), stroke (definite or probable), and HF.
Nguyen 2023 (22)	BMC medical research methodology	Young adults aged 18-30 years at enrollment (CARDIA)	United States	Community	Maximum: 17-year	CVD	3,539	Mean in year: 40 (SD 3.6)	66.0%	5.0%	Incident CVD event included CHD (MI, acute coronary syndrome, or CHD death, including fatal MI), stroke, transient ischemic attack, hospitalization for HF, intervention for peripheral arterial disease, or death from cardiovascular causes.

Qian 2023 (23)	BMC public health	People aged 30-74 years free of ASCVD at baseline	China	Communit y	Median in year: 5.79	ASCVD	7,975 (4,054 men; 3,920 women)	Men (mean (SD)) in year: 44.08(10.85). Women (mean (SD)) in year: 43.31(10.38)	49.0%	10.19% (7.57% in men and 14.44% in women)	ASCVD was diagnosed as nonfatal acute MI, death from CHD, or fatal or nonfatal stroke.
Ren 2022 (24)	Frontiers in Cardiovasc ular Medicine	Adults with diabetic kidney disease with no history of CVD or coronary revascularization (administrative data)	China	Institution	Medium (IQR) in month: 10.4 (3.8–23.4)	CVD	890	Median (IQR) in year: 52 (45-60)	62.6%	31.91%	First occurrence of a subsequent CVD, including CHD, MI, angina, and coronary revascularization); cerebrovascular disease (hemorrhagic stroke and ischaemic stroke); congestive heart failure and peripheral arterial disease (amputations, aortic aneurysm, revascularization of the aorta or other peripheral arteries) and the combination of cardiovascular events.
Rigdon 2019 (25)	BMJ Open	Adults aged 20–79 years with no prior CVD history (NHANES)	United States	Communit y	Median in year: 6.6	CVD mortality	41,990	Mean in year: 50	53%	4.0%	CVD mortality was defined as death from heart disease or cerebrovascular diseases.
Sabovcik 2022 (26)	Frontiers in Cardiovasc ular Medicine	Adults aged 30-80 years without HF at baseline (HOMAGE meta-data)	Multiple countries (United Kingdom, Ireland, Nordic countries, Belgium United states, Netherlands, Scotland, Ireland, and Italy)	Communit y	Median (IQR) in year: 5.40 (4.28–6.52)	Incident non-fatal HF hospitalisation	30,354	Mean (SD) in year: 66 (9)	33.43%	3.52%	Incident non-fatal HF was defined as HF hospitalisation
Segar 2019 (27)	Diabetes care	Adults (aged 40-70 years) with type 2 diabetes mellitus who had no history of prevalent HF at baseline (ACCORD trial)	United States and Canada	Institution	Median in year: 4.9	Hospitalization or death due to HF	8,756	Mean (SD) in year: 62.7 (6.6)	38.5%	3.6%	Hospitalisation for HF was based on documented clinical and radiologic evidence of clinical HF and congestion. Death due to HF or cardiogenic shock was defined as a death with clinical, radiologic, or postmortem evidence of HF, in the absence of acute ischemic event.
Segar 2021 (28)	Circulation	Participants aged >40 years and free of HF at baseline	United States	Communit y	10-year	HF	Black adults: 4,141; for external	Mean (SD) in year (Black adults: 58.1 (10.5)-62.4	Black adults: 56.8%-64.5%.	Black adults (7.0%; men: 6.7%), women: 7.1%). White	Incident HF events were identified by the first hospitalisation event with HF

		(ARIC, DHS, JHS, MESA)					validation for Black adults: 3,845. White adults: 7,858; for external validation: 3,236.	(5.9). White adults: 60.2 (10.8)-63.0 (5.6))	White adults: 52.4%-53.8%).	adults (8.1%; men: 9.4%, women: 7.0%). In the external validation cohort, Black adults: 7.4%; and White adults: 3.1%.	
Stabellini 2023 (29)	Cancers	Women aged ≥ 18 years who are diagnosed with breast cancer at any stage (administrative data)	United States	Institution	Median (IQR) in month: 5.8 (1.5-13.62)	MACE	4,309	Median (IQR) in year: 63 (53-72)	100%	11.4%	MACE included HF, acute coronary syndrome, atrial fibrillation, and ischemic stroke.
Sung 2019 (30)	PLoS ONE	Adults (age 40 to 79 years) who did not have CVD at the baseline (administrative data)	Republic of Korea	Community	Maximum: 10-years	CVD	361,239 External validation set:4,292	Mean (SD) in year: 51.2 (8.9)	43.24%	7.0%	CVD were defined as CVD mortality (International Classification of Diseases 10th edition (ICD-10) code), hospitalisation due to MI, coronary arterial intervention or bypass surgery or hospitalization due to stroke
Turchin 2023 (31)	Obesity Science and Practice	Adults (age 18-80 years) with body mass index (BMI) between 25 and 80 kg/m2 who were being treated in primary care practices (administrative data)	United States	Institution	Median in year: 5.6 years	ASCVD and HF	433,272	Mean (SD) in year: 47.9 (15.7)	52.2%	ASCVD: 11.7%; and HF: 5.0%	Based on International Classification of Diseases 9th and 10th edition (ICD-9 and ICD-10.
Wang 2023 (32)	International Journal of Cardiology	Adults (aged ≥45 years) recruited randomly from the general population (45 and Up study)	Australia	Community	Median in year (CVD mortality: 10.4; and IHD hospitalisation: 11.6)	CVD mortality and IHD-related hospitalisation	187,268	Mean (SD) in years (CVD mortality- No: 59.4 (9.6); Yes: 76.5 (10.6). IHD hospitalisation- No: 59.5 (9.8); Yes: 64.1 (9.9))	CVD Mortality - No: (57.2%); Yes: (47.5%). IHD hospitalisation- No: (58.4%); Yes: (38.0%)	CVD mortality: 2.0%; and IHD hospitalisation: 6.9%.	CVD mortality: CVD-related mortality (ICD codes I00–I99, G45 and G46). IHD hospitalisation was defined as a primary diagnosis with ICD codes I20-I25, also known as CHD, coronary artery disease and atherosclerotic heart disease
Zhuang 2022 (33)	The Canadian Journal of Cardiology	Middle-aged (age 45 to 64) adults (ARIC)	United States	Community	Median in year (CVD: 23.36; and CHD: 25.03).	CVD and CHD	14,842	Mean (SD) in year: 54.2 (5.8)	54.8%	CVD: 32.3%; and CHD: 16.4%.	CVD represented a composite of CHD, stroke, and HF. The criteria for CHD included any MI, resuscitated cardiac arrest,

											definite angina, probable angina followed by revascularization, and CHD mortality.

Abbreviations- ASCVD: Atherosclerotic cardiovascular disease; CAD: Coronary artery disease; CHD: Coronary heart disease; CVD: Cardiovascular diseas e; HF: Heart failure; IQR: Inter quartile range; MACE: Major cardiovascular accident; MI: Myocardial infarction; NR: Not reported; and SD: Standard deviation.

Table S5. Characteristics of survival prediction models used for cardiovascular disease risk prediction.

Author and Year	Modeling methods		Best performed model	Performance measures	Values of the importance measures with 95%CI, if any, (for the best fitted model)	Number of predictors		Methods to select final predictors	Types of candidate predictors	Stratification based on gender	Accounted SDoH variables	Model validation	Model interpretation techniques (Explainable AI)
	Machine learning algorithms	Deep learning algorithms				Candidate	Final						
Ambale-Venkatesh 2017 (1)	LASSO-Cox and RSF.<br>- standard Cox-PH model for comparison	None	RSF	C-index and Brier score	C-index-CVD: 0.80; CHD: 0.80; Stroke: 0.75; HF: 0.84.<br>Brier score-CVD: 0.079; CHD: 0.065; Stroke: 0.030; HF: 0.033.	735 (3 SDoH)	20 (0 SDoH)	RSF based on minimal depth of the maximal subtree	Traditional risk factors and imaging features	No	Level of education, economic status/ income, and race	Internal: Train-test split<br>External: No	None
Barbieri 2022 (2)	None.<br>- standard Cox-PH model for comparison	DeepSurv	DeepSurv	C-index and integrated Brier score	Women (C-index: 0.813 (0.812, 0.814), integrated Brier score: 0.00971 (0.00970, 0.00972)).<br>Men (C-index: 0.771 (0.771, 0.772), integrated Brier score: 0.0176 (0.0176, 0.0176).	23 (2 SDoH)	23 (2 SDoH)	Selection not done	Traditional factors and all diagnoses, procedures, and medications	Yes	*Ethnicity and level of deprivation*	Internal: Stratified 5 × 2 cross-validation<br>External: No	None
Bauer 2023 (3)	RSF.<br>- standard Cox-PH model for comparison	None	RSF	C-index	C-index: 0.74(0.71, 0.76)	18 (0 SDoH)	18 (0 SDoH)	Selection not done	Traditional factors, and Cardiac Computed Tomography Angiography derived variables (imaging feature)	No	None	Internal: Repeated nested cross validation<br>External: No	Permutation feature importance
Blanchard 2022 (4)	None.<br>- standard Cox-PH model for comparison	Deep survival conventional neural network	Deep survival conventional neural network	C-index and AUC	Whole sample: C-index: 0.788 and AUC: 0.823<br>Women: C-index: 0.792 and AUC: 0.821<br>Men: C-index: 0.742 and AUC: 0.779	12 (0 SDoH)	12 (0 SDoH)	Selection not done	Traditional factors and sleep signals	Yes	None	Internal: Train-test split<br>External: No	Contribution of features using weighted ratio (i.e., importance of the sleep signals compared to the clinical feature)
Brester 2023 (5)	RSF.<br>- standard cause-specific and sub-distribution	None	RSF	AUC	NR	950	613	Selection not done (considered variables with less than 5%	No detailed information given (majorly traditional factors)	Not necessary (All men)	Probably none (Not clear because list of variables is not given)	Internal: train-test split<br>External: No	None

	PH models for comparison							missing values)					
Chhoa 2023 (6)	Elastic Net Cox, Component-Wise Gradient Boosting, and RSF	None	RSF (based on discrimination) and Component-Wise Gradient Boosting model (according to integrated brier score)	Brier score and AUC	Component-Wise Gradient Boosting model (Brier Score: 0.165(SE=0.022). RSF (AUC: 0.764 (SE=0.068)	99 (1 SDoH)	99 (1 SDoH)	Selection not done	Traditional factors, medical history and associated pathology, disease history, MRI features, Genetic information, biological sampling, and Clinical and cognitive/neuropsychological scores	No	*Level of education*	Internal: nested cross-validation External: No	Using component wise gradient boosting coefficients
Chun 2021 (7)	RSF. - Framingham Stroke Risk Profile and standard Cox-PH model for comparison	None	RSF	AUC and Nam-D'Agostino test (for calibration)	Men: AUC: 0.826 (0.818, 0.834), Nam-D'Agostino test (x^2: 61(36, 90)). Women: AUC: 0.832 (0.824, 0.839), Nam-D'Agostino test (x^2: 62 (36, 95))	143 (Categorical variables were dummy coded) (4 SDoH)	143 (4 SDoH)	Selection not done	Sociodemographic factors, diet, medical history, physical activity, physical measurements, and traditional factors.	Yes	*Region, level of education, occupation, and income*	Internal: train-test split External: No	Feature importance (Gini importance)
Deng 2023 (8)	None. -Standard Cox-PH model and Pooled Cohort Equations (PCE) for comparison	Nnet-survival, Deepsurv, and Cox-nnet	PCE for Black men. DeepSurv for Black women and White men and Women.	C-index	10x10 CV -C-index (for White men: 0.7371; White women: 0.797; Black men: 0.698; and Black women: 0.789).	7 (0 SDoH)	7 (0 SDoH)	Selection not done	Traditional factors	Yes (along with race – Black/ White)	*Race (as a stratified variable)*	Internal: 10x10 cross-validation External: Yes	None
Duan 2024* (9)	Elastic Net Cox, RSF, Survival	None	Elastic Net Cox	C-index, AUC, and	C-index: 0.926 (0.924, 0.927); AUC: 0.935 (0.933,0.936);	61 (4 SDoH)	38 (4 SDoH)	Elastic Net	Traditional factors, and	No	*Ethnicity, level of education, marital status, and family*	Internal: train-test split External: No	Shapley Additive exPlanations

	Gradient Boosting, and ExtraSurvival Trees.<br>- Standard Cox-PH model for comparison			Brier score	Brier score: 0.024 (0.023,0.025).			Penalised Cox-PH model	environmental chemicals		*income-poverty ratio level*		(SHAP), and partial dependence plots
Farhadian 2021 (10)	RSF.<br>- Standard Cox-PH model for comparison	None	RSF	C index and integrated Brier score	C index: 0.648; integrated Brier score: 0.124	13 (0 SDoH)	13 (0 SDoH)	Selection not done	Traditional factors	No	None	Internal: Out-of-bag (OBB) sample<br>External: No	Permutation feature importance
Feng 2022 (11)	LMTLR and RSF.<br>- Standard Cox-PH model for comparison	NMTLR	NMTLR	C-index, Brier score, RMSE, and MAE	C-index: 0.8202; Brier score: 0.0243; RMSE: 143.49; and MAE: 132.54.	25 (1 SDoH)	25 (1 SDoH)	Selection not done	Traditional factors	No	*Region of residence (urban vs rural)*	Internal: train-test split<br>External: No	None
Gandin 2023 (12)	None.<br>- Standard Cox-PH model for comparison	DeepSurv	DeepSurv	C-index, AUC, graphical assessment of calibration, and Integrated Calibration Index (ICI).	C-index: 0.768; 5-year AUC: 0.780 (0.743,0.817); and 5-year ICI: 0.015	33 (0 SDoH)	20 (0 SDoH)	A forward selection procedure using c-index	Traditional factors, laboratory tests and procedures, and cardiovascular drugs prescriptions, and comorbidities	No	None	Internal: train-test split<br>External: No	Partial dependence plots (PDPs)
Gao 2023 (13)	Elastic Net Cox, Survival Gradient Boosting, FastKernelSuvSVM, FastSurvSVM, and RSF.<br>- Standard Cox-PH model for comparison	Denoising autoencoder Survival network: a deep learning model	Denoising autoencoder Survival network	C-index	C-index: 0.846 (0.790, 0.888)	36 (0 SDoH)	36 (0 SDoH)	Selection not done	Heart motion information and traditional factors	No	None	Internal: Bootstrap method<br>External: No	None
Garcia-Carretero 2019 (14)	LASSO-Cox and Elastic Net Cox. - Standard Cox-PH model for comparison	None	Elastic Net Cox	C-index, AUC, and calibration plot	C-index: 0.658 and AUC: 0.673	15 (0 SDoH)	3 (0 SDoH)	LASSO and Elastic Net Penalised Cox-PH model	Traditional factors	No	None	Internal: 10-fold cross-validation<br>External: No	Nomogram

Hathaway 2021 (15)	RSF and linear SVM.<br>- Standard Cox-PH model for comparison	NMTLR and DeepSurv.	DeepSurv	C-index, AUC, net reclassification improvement, and integrated Brier score	C-index: 0.80 (0.78, 0.82); AUC: 0.84 (0.83–0.84); and integrated Brier score; 0.09 (0.08, 0.09).	37 (3 SDoH)	33 (3 SDoH)	Selection not done but features with correlation coefficient >0.8 (n=4) were removed.	Traditional factors, inflammatory biomarkers, and imaging features	No	*Level of education, income, and race/ethnicity*	Internal: train-test split<br>External: Yes	RSF: Feature importance (using mean decrease Gini).<br>Others: Permutation feature importance
Jain 2021 (16)	Extreme Gradient Boosting with a Cox loss function.	None	Extreme gradient boosting	C-index	C-index: 0.72 (0.59, 0.85)	35 (1 SDoH)	35 (1 SDoH)	Selection not done	Traditional factors, prior cardiac conditions, indication for liver Transplantation and relevant laboratory values	No	*Race*	Internal: 5-fold cross-validation<br>External: No	Shapley Additive exPlanations (SHAP)
Kim 2023 (17)	RSF.<br>- Standard Cox-PH model for comparison	DeepSurv and Deep Survival Machines (DeepSM)	DeepSurv	C- index and integrated Brier score	Without brain-MRI (diffusion-weighted imaging – DWI) features: C-index: 0.824 (0.750, 0.885); integrated Brier score: 0.066 (0.051-0.083)<br>With DWI: C-index 0.850 (0.784-0.904); IBS: 0.064 (0.048-0.081)	60 (0 SDoH)	39 (0 SDoH)	LASSO Penalised Cox-PH model	Traditional factors and imaging features	No	None	Internal: train-test split<br>External: No	Permutation feature importance
Lin 2023 (18)	LASSO-Cox model and RSF	DeepSurv	DeepSurv	C-index	C-index: 0.826	25 (0 SDoH)	10 (0 SDoH)	Selection not done but used permutation-based feature importance from RSF and LASSO penalized Cox-PH model i.e., analysed top 5, 10, 15, 20 and 25 features.	Traditional factors and vital sign values	No	None	Internal: train-test split<br>External: No	Coefficient values for and then simplified risk scoring system for LASSO-Cox, and Permutation feature importance for RSF

Mauger 2023 (19)	RSF	None	RSF	IPA and AUC.	CVD: IPA (%): 12.7 ± (1.2) and AUC: 0.78 ± 0.00.<br>CHD: IPA (%): 11.5 ± 0.8 ± 1.2 and AUC: 0.77 ± 0.01.<br>HF: IPA (%): 14.6 ± 2.4 and AUC: 0.83 ± 0.01	46 (1 SDoH)	46 (1 SDoH)	Selection not done	Traditional factors and image features	No	*Race*	Internal: train-test split<br>External: No	Feature importance (mean of the minimal depth of the maximal subtree)
Moreno-Sánchez 2023 (20)	RSF, Extra Survival Trees, Survival Gradient Boosting, and Survival support vector machines (SSVMs).<br>- Standard Cox-PH model for comparison	None	Gradient Boosting models	C-index and AUC	C-index: 0.724 and AUC: 0.748	11 (0 SDoH)	7 (0 SDoH)	ANOVA, chi-squared, Mutual information (mut-inf), or recursive feature elimination (RFE)	Traditional factors	No	None	Internal: train-test split<br>External: No	Shapley Additive exPlanations (SHAP) and Partial dependence plots (PDPs)
Morris 2023 (21)	RSF and Ridge regression	DeepHit	DeepHit	C-index	Traditional RFs - C-index: 0.76<br>Traditional RFs + Psychosocial/socioeconomic - C-index: 0.76<br>Traditional RFs + Psychosocial/socioeconomic + Environmental - C-index: 0.76	161 (Categorical variables were dummy coded) (14 SDoH; summarised accordingly)	161 (14 SDoH; summarised accordingly)	Selection not done	Traditional and social determinants (psychosocial, socioeconomic, and environmental factors)	No	*Health insurance, discrimination, favorable food stores, family income, Stress, employment status, Proportion of households in census tract with no vehicle, walking destinations available within area to resident, race, depressive Symptoms, level of education, Unconditional Empirical, Bayes Estimate for Social Cohesion PCA-base, and occupation, country of birth*	Internal: train-test split<br>External: No	Shapley Additive Explanation (SHAP)
Nguyen 2023 (22)	LASSO-Cox and RSF.<br>- Standard Cox-PH model for comparison	DeepHit	RSF	C-index, integrated AUC, and Brier score	C-index: 0.778 (0.757, 0.801); integrated AUC: 0.808 (0.790, 0.826); and Brier score: Lower Brier Score (its exact value not reported)	35 (3 SDoH)	35 (3 SDoH)	Selection not done	Traditional risk factors, anthropometry, physiological measures, medications, socioeconomi	No	*Race, level of education, and ability to pay for the very basics*	Internal: 5-fold x 2 times cross-validation<br>External: No	Permutation feature importance, Shapley Additive Explanation (SHAP), and Temporal

									c, and medical history				Importance Model Explanation (TIME)
Qian 2023 (23)	LASSO-Cox and RSF.<br>- Standard Cox-PH model, China-PAR, and Framingham risk score models for comparison	None	RSF	C-index, AUC, and Brier score	Men: C-index: 0.780 (0.730, 0.829); AUC: 0.791 (0.767,0.813); and Brier Score: 0.060.<br>Women: C-index: 0.737 (0.702,0.771); AUC: 0.759 (0.734,0.783); and Brier Score: 0.110.	61 (3 SDoH)	20 (0 SDoH) in men and 18 (0 SDoH) in women	Cox multivariate analysis, LASSO-Cox, and RSF	Traditional factors, serological indicators, and questionnaire information	Yes	Level of education, occupation, and marital status	Internal: train-test split<br>External: No	Permutation feature importance
Ren 2022 (24)	RSF.<br>- Standard Cox-PH model for comparison	DeepSurv	DeepSurv	C-index, AUC, and integrated Brier score	C-index: 0.767(0.717, 0.817); AUC: 0.780 (0.721, 0.839), and integrated Brier score: 0.067.	91 (uncertain SDoH)	7 (0 SDoH)	LASSO Penalised Cox-PH model	Demographic, clinical characteristics , and laboratory results	No	No (in the final model) and uncertain at preprocessing stage	Internal: train-test split<br>External: No	Feature importance (calculated by their component weights)
Rigdon 2019 (25)	Survival Gradient Boosting and RSF.<br>- Standard Cox-PH model for comparison	None	RSF	C-index and Greenwood-Nam-D'Agostino test a (for calibration)	With SDoH: C-index: 0.93 (NR); and Calibration slope: 1.01 (NR).<br>Without SDoH: C-index: 0.93 (0.92, 0.94) and calibration slope: 1.01 (0.76 to 1.27	11 traditional risk factors and 107 nutritional variables (3 SDoH)	Same 128 (3 SDoH)	Selection not done	Traditional factors and nutrition related variables	No	*Level of education, poverty, race/ethnicity*	Internal: train-test split<br>External: No	Partial dependence plots (PDPs)
Sabovcik 2022 (26)	Survival Gradient Boosting, Elastic Net Cox, and stacking method.<br>- Pooled Cohort Equations to Prevent HF (PCP-HF) score for comparison	None	Survival Gradient Boosting	C-index and calibration	C-index: 0.735 (0.728, 0.742; and Calibration: well calibrated	33 (0 SDoH)	33 (0 SDoH)	Selection not done	Traditional factors and Electrocardiographic parameters	No	None	Internal: train-test split<br>External: Yes	Permutation based feature importance
Segar 2019 (27)	RSF.<br>- Standard Cox-PH model for comparison	None	RSF	C-index and Hosmer and	C-index: 0.77 (0.75, 0.80); and Hosmer-Lemshow statistic $x^{2:}$ 5 9.63, P=0.29	109 (3 SDoH)	8-11 (0 SDoH)	11 (0 SDoH) from stepwise backward;	Traditional factors, Electrocardiographic parameters,	No	Race, level of education, and living with other adults	Internal: train-test split<br>External: Yes	Machine learning–derived risk score

				Lemshow test	- A model with RSF-selected variables performed better.			8 (0 SDoH) from stepwise forward; 10 (0 SDoH) from permutation based RSF	baseline antihyperglycemic therapies, and treatment randomisation				
Segar 2021 (28)	LASSO-Cox, Ridge-Cox, Oblique RSF, Gradient Boosting, - Standard Cox-PH model for comparison	None	Oblique RSF	C-index	Race-specific model: C-index: 0.88 (0.85, 0.90) among Black adults and 0.88 (0.85, 0.90) for White adults. Race as a covariate model: C-index 0.81 (0.78-0.83) for Black adults; C-index 0.80 (0.76-0.85) for White adults.	54 [5 SDoH SP] (remained 39 [3 SDoH] after excluding the variables with >20% missingness and correlation coefficient >0.70	39 vs 20 (3 SDoH)	Selection not done (but compared the performance of the RSF with 20 variables and the 39 variables based on C-index and found RSF model with 20 variables a relatively better model)	Traditional factors, electrocardiographic parameters, and medications	No	*Income level*, family income, *level of education*, time caring for others, and *race as a stratified variable*	Internal: train-test split External: Yes	Permutation-based feature importance
Stabellini 2023 (29)	Extreme Gradient Boosting	None	Extreme Gradient Boosting (no comparison)	C-index	C-index: 0.78 (0.76, 0.79) and 0.81(0.80, 0.82) without and with SDoH data, respectively for the race-agnostic models. C-index: 0.74 (0.72, 0.76) and 0.75 (0.73, 0.78) in non-Hispanic Black women models without and with SDoH data, respectively. C-index: 0.79 (0.77, 0.80) and 0.79 (0.77, 0.80) among non-Hispanic White women models without and with SDOH data, respectively.	39 (24 SDoH)	39 (24 SDoH)	Selection not done	Traditional factors, SDoH, tumor characteristics, and breast cancer treatment	Not necessary (All women)	*Social and community context (marital status, number of household members, distance to closest relatives); economic stability (address stability, property status, annual income, properties owned, wealth index, household income, total count of transport properties owned); neighborhood and built environment (crime index, burglary index, car theft index, murder index, neighborhood median household income, neighborhood median*	Internal: train-test split External: No	None

											home values); and educational access and quality (education institution rating, college attendance).		
Sung 2019 (30)	None.<br>- Standard Cox-PH model for comparison	Deep learning algorithm model based on survival analysis (Recurrent Neural Network Long Short-Term Memory (RNN-LSTM))	Deep learnin g model	C-index or AUC.	Men: 2-year AUC: 0.94 (0.91, 0.97).<br>Women: AUC: 0.96 (0.95, 0.97)	23 (0 SDoH)	23 (0 SDoH)	Selection not done	Traditional factors	Yes	None	Internal: train-test split<br>External: Yes	Layer-wise Relevance Propagation (LRP)
Turchin 2023 (31)	LASSO-Cox and RSF	None	RSF	C-index	ASCVD: C-index: 0.812.<br>HF: C-index: 0.871.	40	35 (2 SDoH) for ASCV D<br>32 (2 SDoH) for HF	Bivariate analysis with outcome and selected the candidate variables with P <0.15 and RSF model was conducted using the minimal depth approach	Traditional factors	No	*Marital status and commercial insurance*	Internal: train-test split<br>External: No	None
Wang 2023 (32)	LASSO-Cox, Ridge regression, fast survival SVM, RSF	None	LASS O-Cox	C-index (Harrel's and Uno's)	CVD Mortality: Harrel's C-index: 0.9004; Uno's C-index: 0.8976<br>IHD Hospitalisation: Harrel's C-index: 0.7178; Uno's C-index: 0.7105	98 (7 SDoH)	98 (7 SDoH) (3 SDoH in top 20 of CVD mortali ty model) (2 SDoH in IHD hospita	Selection not done	Socioeconomi c status, traditional factors, and dietary patterns	No	*Health insurance*, level of education, country of birth, IRSD quintile, remoteness, *Annual household income*, and *employment*	Internal: train-test split<br>External: No	SHapley Additive exPlanations (SHAP)

							lisation top 20)					
Zhuang 2022 (33)	LASSO-Cox and RSF.<br>- Standard Cox-PH model for comparison	None	RSF	C-index and Brier score	CVD: C-index: 0.78 (0.77, 0.78; Brier score: 0.059. CHD: C-index: 0.80 (0.79, 0.81); Brier score: 0.032.	300	20 (1 SDoH in CVD, 0 SDoH in CHD)	RSF (Based on minimal depth of the maximal subtree)) – top 20 for each outcome	Traditional factors, laboratory biomarkers, family history, and imaging/electr ocardiographi c variables	No	Race, level of education, and *income*	Internal: Stratified 5-fold cross-validation<br>External: No	Feature importance (mean of the minimal depth of the maximal subtree)

Abbreviation- ASCVD: Atherosclerotic cardiovascular disease; AUC: Area under the curve; CHD: Coronary heart disease; CI: Confidence interval; C-index: Concordance index; Cox-PH: Cox Proportional Hazard; CV: Cross validation; CVD: Cardiovascular disease; HF: Heart failure; ICI: Integrated calibration index; IPA: Index of prediction accuracy; LASSO: Least Absolut Shrinkage and Selection Operator; MAE: Mean absolute error; NMTLR: Neural Multi-Task Logistic Regression; NR: Not reported; RMSE: Root mean square error; RSF: Random survival forest; SDoH: Social determinants of health; and SE: Standard error.
*This study was accepted (pre-proof) during our search period and become published in January 2024.

Table S6. Patient recruitment year, missing data management, hyperparameter tuning, and software (including libraries/packages) utilised.

Author and Year	Patient recruitment year	Missing data management methods	Hyperparameter tunning methods	Software*	Packages/libraries utilised for training machine learning models	Code/source code
Ambale-Venkatesh 2017 (1)	2000–2002	Adaptive tree imputation method	NR	R (version NR)	NR	NR
Barbieri 2022 (2)	2012	Complete case analysis	Tree-structured Parzen Estimator	Python version 3.7.5	PyTorch, PyCox library, and Optuna	https://github.com/VIEW2020/Varianz2012
Bauer 2023 (3)	2004-2017	No missing data	Grid search	R version 3.6.1, Python version 3.7.3), and MATLAB R2019a	scikit-survival	https://github.com/DHM-CCTA-ML/CCTA_ML_TimeToEvent
Blanchard 2022 (4)	2007-2018	Mode for categorical features and MICE for continuous features	5-folds cross validation (to select model architecture)	Python (version NR)	Tensorflow and Scikit Survival	NR
Brester 2023 (5)	1984- 1989	MICE	NR	R and Python (versions NR)	randomForestSRC and see the code for others	https://github.com/christinabrester/isMode
Chhoa 2023 (6)	2003-2020	Features with ≥ 50% missing values were discarded. The remaining handled using mode and median imputation	Nested cross-validation	Python (version NR)	scikit-survival	NR
Chun 2021 (7)	2004–2008	Mean imputation	Grid search	Python version 3.7.0 and R version 3.6.1	glmnet, ranger, keras	NR
Deng 2023 (8)	1948 -2010	Complete case analysis	Grid search	Python, version 3.7.3 and R, version 3.6.0	scikit-learn, PyTorch	NR
Duan 2024 (9)	2003-2018	MissForest	Grid search	Stata SE 15.1, R 4.0.5, and Python 3.11.2	Scikit-survival	NR
Farhadian 2021 (10)	2009-2012	No missing data	NR	R version 3.6.3	randomForestSRC, pec, survival	NR
Feng 2022 (11)	2009-2015	Complete case analysis	NR	SAS version 9.4, R version 3.5.1, and Python version 3.7.6.	PySurvival	NR
Gandin 2023 (12)	2009-2018	Mean	NR	R version 4.2.1 and Python 3.8.10	PyTorch	NR
Gao 2023 (13)	2015-2020	MICE	NR	SPSS 23.0 and R version 3.6.3	NR	NR
Garcia-Carretero 2019 (14)	2006-2017	Complete case analysis	10-fold cross-validation	R version 3.3.3	NR	NR
Hathaway 2021 (15)	200-2002	Median imputation	Not done	R version 3.6.2 and Python 3.7	Pysurvival	https://github.com/qahathaway/MESA

Jain 2021 (16)	2008-2019	k-nearest-neighbor algorithm	Grid search	Python 3.7.6	XGBoos	NR
Kim 2023 (17)	2010-2019	NR	NR	Python (version NR)	scikit-survival	NR
Lin 2023 (18)	2010-2018	MissForest	NR	Python (version NR) and R version 4.0.	scikit-survival, pycox	NR
Mauger 2023 (19)	2000-2002	No missing data	NR	R (version NR)	NR	NR
Moreno-Sánchez 2023 (20)	2015	No missing data	Grid search	Python (version NR)	scikit-survival	https://github.com/petmoreno/Heart_Failure_Predictor
Morris 2023 (21)	2000-2004	Median	Bayesian hyperparameter optimization (using HyperOpt–an open-source library)	Python version 3.8	PyTorch, PyCox, scikit-survival	NR
Nguyen 2023 (22)	1985-1986	Complete case analysis	NR	R and Python (versions NR)	traj, NbClust, JMBayes, rsfsrc (see the code for others)	https://github.com/cloudbopper/anamod , https://github.com/chl8856/Dynamic-DeepHit, and https://github.com/blue-yonder/tsfresh.
Qian 2023 (23)	2016	Mean and mode imputation	5-fold cross validation	SPSS version 26.0, and R version 4.0	NR	NR
Ren 2022 (24)	2013-2020	MICE	Bayesian hyperparameter optimization	R version 4.1.1, SPSS version 26, and Python v.3.7	TensorFlow	https://github.com/jaredleekatzman/DeepSurv
Rigdon 2019 (25)	1999–2000	MICE	Grid search	Stata version 15 and R version 3.6.1	NR	https://github.com/joerigdon/CVD_Prediction
Sabovcik 2022 (26)	NR	Complete case analysis	Tree-structured parzen estimator	Python (version NR)	scikit-survival	https://github.com/hcve/incidence-hf
Segar 2019 (27)	NR	Random forest imputation	NR	R version 3.5.1	randomForestSRC	NR
Segar 2021 (28)	1996-2004	Random forest imputation	NR	R versions 3.5.1 and 3.6.0	obliqueRSF, glmnet, CoxBoost, and xgboost	NR
Stabellini 2023 (29)	2010-2019	Complete case analysis	Randomised search	R version 4.2.2	mlr3 and mlr3proba	NR
Sung 2019 (30)	2002–2003	Multiple imputations by fully conditional specifications	NR	SAS and R (versions NR)	NR	NR
Turchin 2023 (31)	2000- 2019	MissForest and mean imputation	NR	R version 3.6.3	randomForestSRC and glmnet	NR
Wang 2023 (32)	2005–2009	Median or mean values	Grid search	Python version 3.6.	scikit-survival	NR
Zhuang 2022 (33)	1987-1989	Adaptive tree imputation	NR	R version 2.7.2	randomForestSRC and glmnet	NR

Abbreviation- MICE, Multiple Imputation by Chained Equations; NR, Not reported and/or not sure it is done. *Stata and SPSS were not used to train the models; they were used to preprocess the data.

Reference

1. Ambale-Venkatesh B, Yang X, Wu CO, Liu K, Gregory Hundley W, McClelland R, et al. Cardiovascular Event Prediction by Machine Learning: The Multi-Ethnic Study of Atherosclerosis. CIRCULATION RESEARCH. 2017;121(9):1092-101.
2. Barbieri S, Mehta S, Wu B, Bharat C, Poppe K, Jorm L, et al. Predicting cardiovascular risk from national administrative databases using a combined survival analysis and deep learning approach. International Journal of Epidemiology. 2022;51(3):931-44.
3. Bauer MJ, Nano N, Adolf R, Will A, Hendrich E, Martinoff SA, et al. Prognostic Value of Machine Learning-based Time-toEvent Analysis Using Coronary CT Angiography in Patients with Suspected Coronary Artery Disease. Radiology: Cardiothoracic Imaging. 2023;5(2):e220107.
4. Blanchard M, Feuilloy M, Sabil A, Gerves-Pinquie C, Gagnadoux F, Girault JM. A Deep Survival Learning Approach for Cardiovascular Risk Estimation in Patients With Sleep Apnea. IEEE Access. 2022;10:133468-78.
5. Brester C, Tuomainen TP, Voutilainen A, Kauhanen J, Kolehmainen M. Predicting risk of cardiovascular death in the high-dimensional cohort follow-up data in the presence of competing events: a guide for building a modeling pipeline. Biostatistics and Epidemiology. 2023;7(1):e2084704.
6. Chhoa H, Chabriat H, Chevret S, Biard L. Comparison of models for stroke-free survival prediction in patients with CADASIL. SCIENTIFIC REPORTS. 2023;13(1):22443.
7. Chun M, Clarke R, Cairns BJ, Clifton D, Bennett D, Chen Y, et al. Stroke risk prediction using machine learning: a prospective cohort study of 0.5 million Chinese adults. Journal of the American Medical Informatics Association : JAMIA. 2021;28(8):1719-27.
8. Deng Y, Liu L, Jiang H, Peng Y, Wei Y, Zhou Z, et al. Comparison of State-of-the-Art Neural Network Survival Models with the Pooled Cohort Equations for Cardiovascular Disease Risk Prediction. BMC medical research methodology. 2023;23(1):22.
9. Duan S, Wu Y, Zhu J, Wang X, Zhang Y, Gu C, et al. Development of interpretable machine learning models associated with environmental chemicals to predict all-cause and specific-cause mortality : A longitudinal study based on NHANES. Ecotoxicol Environ Saf. 2024;270:115864.
10. Farhadian M, Dehdar Karsidani S, Mozayanimonfared A, Mahjub H. Risk factors associated with major adverse cardiac and cerebrovascular events following percutaneous coronary intervention: a 10-year follow-up comparing random survival forest and Cox proportional-hazards model. BMC Cardiovascular Disorders. 2021;21(1):38.
11. Feng Y, Leung AA, Lu X, Liang Z, Quan H, Walker RL. Personalized prediction of incident hospitalization for cardiovascular disease in patients with hypertension using machine learning. BMC medical research methodology. 2022;22(1):325.
12. Gandin I, Saccani S, Coser A, Scagnetto A, Cappelletto C, Candido R, et al. Deep-learning-based prognostic modeling for incident heart failure in patients with diabetes using electronic health records: A retrospective cohort study. PLoS ONE. 2023;18(2):e0281878.
13. Gao Y, Zhou Z, Zhang B, Guo S, Bo K, Li S, et al. Deep learning-based prognostic model using non-enhanced cardiac cine MRI for outcome prediction in patients with heart failure. European radiology. 2023;33(11):8203-13.
14. Garcia-Carretero R, Barquero-Perez O, Mora-Jimenez I, Soguero-Ruiz C, Goya-Esteban R, Ramos-Lopez J. Identification of clinically relevant features in hypertensive patients using penalized regression: a case study of cardiovascular events. Medical and Biological Engineering and Computing. 2019;57(9):2011-26.
15. Hathaway QA, Yanamala N, Budoff MJ, Sengupta PP, Zeb I. Deep neural survival networks for cardiovascular risk prediction: The Multi-Ethnic Study of Atherosclerosis (MESA). Computers in Biology and Medicine. 2021;139:104983.

16. Jain V, Bansal A, Radakovich N, Sharma V, Khan MZ, Harris K, et al. Machine Learning Models to Predict Major Adverse Cardiovascular Events After Orthotopic Liver Transplantation: A Cohort Study. Journal of Cardiothoracic and Vascular Anesthesia. 2021;35(7):2063-9.
17. Kim D-Y, Choi K-H, Kim J-H, Hong J, Choi S-M, Park M-S, et al. Deep learning-based personalised outcome prediction after acute ischaemic stroke. Journal of neurology, neurosurgery, and psychiatry. 2023;94(5):369-78.
18. Lin C-H, Kuo Y-W, Huang Y-C, Lee M, Huang Y-W, Kuo C-F, et al. Development and Validation of a Novel Score for Predicting Long-Term Mortality after an Acute Ischemic Stroke. INTERNATIONAL JOURNAL OF ENVIRONMENTAL RESEARCH AND PUBLIC HEALTH. 2023;20(4).
19. Mauger CA, Gilbert K, Suinesiaputra A, Bluemke DA, Wu CO, Lima JAC, et al. Multi-Ethnic Study of Atherosclerosis: Relationship between Left Ventricular Shape at Cardiac MRI and 10-year Outcomes. Radiology. 2023;306(2):e220122.
20. Moreno-Sánchez PA. Improvement of a prediction model for heart failure survival through explainable artificial intelligence. Front Cardiovasc Med. 2023;10:1219586.
21. Morris MC, Moradi H, Aslani M, Sims M, Schlundt D, Kouros CD, et al. Predicting incident cardiovascular disease among African-American adults: A deep learning approach to evaluate social determinants of health in the Jackson heart study. PLoS ONE. 2023;18(11):e0294050.
22. Nguyen HT, Vasconcellos HD, Keck K, Reis JP, Lewis CE, Sidney S, et al. Multivariate longitudinal data for survival analysis of cardiovascular event prediction in young adults: insights from a comparative explainable study. BMC medical research methodology. 2023;23(1):23.
23. Qian X, Keerman M, Zhang X, Guo H, He J, Maimaitijiang R, et al. Study on the prediction model of atherosclerotic cardiovascular disease in the rural Xinjiang population based on survival analysis. BMC public health. 2023;23(1):1041.
24. Ren J, Liu D, Li G, Duan J, Dong J, Liu Z. Prediction and Risk Stratification of Cardiovascular Disease in Diabetic Kidney Disease Patients. FRONTIERS IN CARDIOVASCULAR MEDICINE. 2022;9:923549.
25. Rigdon J, Basu S. Machine learning with sparse nutrition data to improve cardiovascular mortality risk prediction in the USA using nationally randomly sampled data. BMJ OPEN. 2019;9(11):e032703.
26. Sabovcik F, Ntalianis E, Cauwenberghs N, Kuznetsova T. Improving predictive performance in incident heart failure using machine learning and multi-center data. FRONTIERS IN CARDIOVASCULAR MEDICINE. 2022;9:1011071.
27. Segar MW, Vaduganathan M, Patel KV, McGuire DK, Butler J, Fonarow GC, et al. Machine Learning to Predict the Risk of Incident Heart Failure Hospitalization Among Patients With Diabetes: The WATCH-DM Risk Score. Diabetes care. 2019;42(12):2298-306.
28. Segar MW, Jaeger BC, Patel KV, Nambi V, Ndumele CE, Correa A, et al. Development and Validation of Machine Learning-Based Race-Specific Models to Predict 10-Year Risk of Heart Failure: A Multicohort Analysis. Circulation. 2021;143(24):2370-83.
29. Stabellini N, Cullen J, Moore JX, Dent S, Sutton AL, Shanahan J, et al. Social Determinants of Health Data Improve the Prediction of Cardiac Outcomes in Females with Breast Cancer. Cancers. 2023;15(18):4630.
30. Sung JM, Cho I-J, Sung D, Kim S, Kim HC, Chae M-H, et al. Development and verification of prediction models for preventing cardiovascular diseases. PLoS ONE. 2019;14(9):e0222809.
31. Turchin A, Morrison FJ, Shubina M, Lipkovich I, Shinde S, Ahmad NN, et al. EXIST: EXamining rIsk of excesS adiposiTy-Machine learning to predict obesity-related complications. Obesity Science and Practice. 2023.
32. Wang H, Tucker WJ, Jonnagaddala J, Schutte AE, Jalaludin B, Liaw S-T, et al. Using machine learning to predict cardiovascular risk using self-reported questionnaires: Findings from the 45 and Up Study. INTERNATIONAL JOURNAL OF CARDIOLOGY. 2023;386:149-56.

33. Zhuang X-D, Tian T, Liao L-Z, Dong Y-H, Zhou H-J, Zhang S-Z, et al. Deep Phenotyping and Prediction of Long-term Cardiovascular Disease: Optimized by Machine Learning. The Canadian journal of cardiology. 2022;38(6):774-82.
